# A relictual troglomorphic harvestman discovered in a volcanic cave of western Argentina: *Otilioleptes marcelae*, new genus, new species, and Otilioleptidae, new family (Arachnida, Opiliones, Gonyleptoidea)

**DOI:** 10.1371/journal.pone.0223828

**Published:** 2019-10-23

**Authors:** Luis E. Acosta

**Affiliations:** 1 Universidad Nacional de Córdoba, Facultad de Ciencias Exactas, Físicas y Naturales, Cátedra de Diversidad Biológica II, Córdoba, Argentina; 2 Consejo Nacional de Investigaciones Científicas y Técnicas (CONICET), Instituto de Diversidad y Ecología Animal (IDEA), Córdoba, Argentina; Natural History Museum of London, UNITED KINGDOM

## Abstract

The troglomorphic harvestman *Otilioleptes marcelae* gen. nov., sp. nov. from the basaltic cave Doña Otilia, Payunia region, Mendoza Province, Argentina, is described. Its systematic affinities were studied through cladistic and Bayesian analyses that included representatives of Gonyleptoidea; it was determined to represent a new monotypic family, Otilioleptidae fam. nov., occupying a basal position within the clade Laminata. This species shows accentuated troglomorphic traits, typical for troglobitic harvestmen: elongated appendages, depigmentation, reduction of eyes and fading of scutal sulci. Additionally, it almost lacks sexual dimorphism, the distal portion of coxa IV is not completely fused to the stigmatic segment, and penis morphology is remarkably divergent with other Laminata; these features cannot be attributed to cave adaptation and may reflect early lineage divergence. *Otilioleptes marcelae* is the first troglobitic gonyleptoid known from a lava tube. The xeric environments around the cave (Patagonian ecoregion) and the paleoenvironmental history of the area suggest the relictual character of *O*. *marcelae*. Scattered evidence supports a long time evolutionary scenario and a presumable relationship with the Chilean opiliofauna (especially with genus *Osornogyndes*). A comparative overview of all known troglobitic gonyleptoids is provided. The urgent need to protect this new species and its unique cave environment is emphasized.

## Introduction

Lava tubes, i.e., caves of volcanic origin, are relatively young formations with the same age as the igneous rock in which they are built [1, 2]. In this feature, they differ from the better-studied karst caves, which undergo a continuous process of development and growth, through the dissolution of carbonates (limestone, gypsum) over an extended geological time. In both cases, the presence of obligate cave dwellers (troglobites = troglobionts) has always stimulated the interest of evolutionary researchers. In the Neotropics, several harvestmen are known to be troglobites, but up to now, all were recorded from karst or sandstone caves. This paper reports the finding of a highly specialized harvestman in a basaltic cave of western Argentina (Cueva Doña Otilia), which is described below as *Otilioleptes marcelae* gen. nov., sp. nov. (Opiliones, Laniatores, Gonyleptoidea). This hypogean species is the very first gonyleptoid known from inside a lava tube, and the first true troglobitic gonyleptoid reported from Argentina. Behind *Picunchenops spelaeus* Maury, 1988 (Laniatores, Triaenonychoidea, Triaenonychidae) [3], it is the second troglobitic harvestman known in that country.

Cave harvestmen, especially those placed in the middle of a hostile epigean environment, might reveal a significant meaning from a biogeographical and evolutionary perspective, as presumed relics of ancient distributional patterns or lineages [4–6]. Doña Otilia cave is located in an extensive xeric region, the Andean and sub-Andean domains in central-western Argentina, where the existence of epigean harvestmen is almost inconceivable [5]. The low precipitation rate, below 400 mm/yr, was deemed to be the cause of the presumed complete lack of harvestmen in that extensive area [7, 8]. Only a few isolated populations were discovered in some sites, scattered across this vast region, otherwise “negative” for harvestmen [4, 5, 9–12]. Three of them were found in caverns: the mentioned *P*. *spelaeus* (Triaenonychidae), from caves of the Cuchillo Curá system, Neuquén Province, and two unidentified members of *“Parabalta”* Roewer, 1913 (Gonyleptidae), from the Caverna de Chorriaca, Neuquén Province and the renowned Caverna de Las Brujas, Mendoza Province [3, 4]. The unnamed harvestman from Las Brujas has become a kind of “flagship” among Argentinean speleologists, but so far, *Picunchenops spelaeus* was strictly the only true troglobitic member of the order in this country. Las Brujas is one of many caves occurring in the vicinity of Malargüe in southern Mendoza Province. They have been actively surveyed since decades ago [13], including several lava tubes placed in the large basaltic unit known as “Payunia” or “Payenia”–among them, Doña Otilia cave. This cavity has been reported to harbor an interesting invertebrate fauna, thus revealing its biospeleological potential [14]. Harvestmen caught in this cave are primarily the result of the collecting efforts of Marcela Peralta in Doña Otilia, who kindly sent me this odd new gonyleptoid for study.

**Fig 1:**
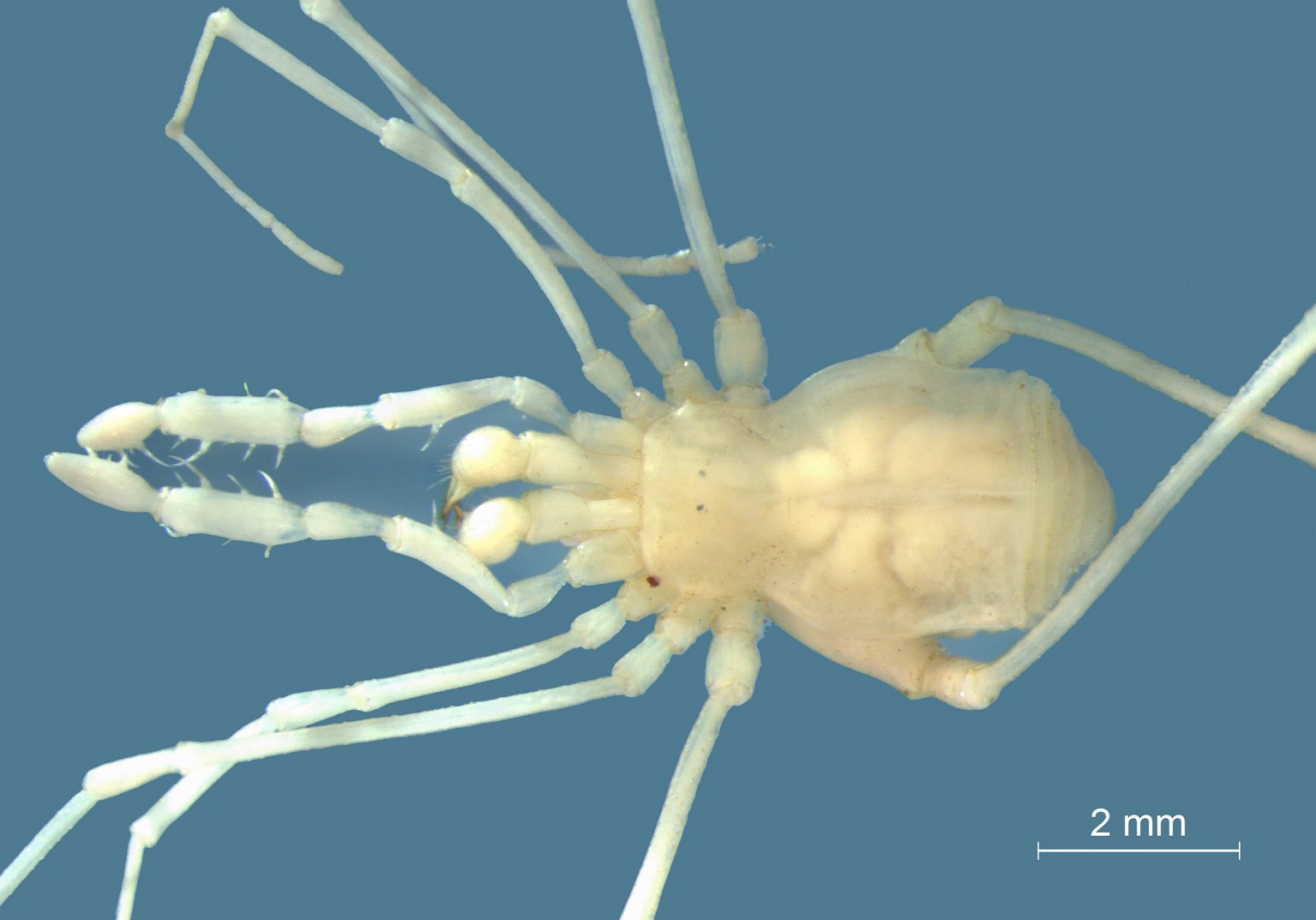
*Otilioleptes marcelae* gen. nov., sp. nov. Paratype male (FML-OPIL 00218), dorsal view. Photo: Abel Pérez-González.

The morphology of *Otilioleptes marcelae* revealed well-defined troglomorphic traits: depigmentation, weak tegumentary sclerotization, extreme reduction of eyes, elongation of appendages, fading of scutal grooves, and enlargement of pedipalp spines (Fig 1). All those features are typical for cave-adapted harvestmen [3, 4, 15–21] and strongly suggest its condition as a true troglobite, i.e., a species confined into the cave through its entire life cycle [2, 22]. Aside from the extreme simplification of external traits shown by *O*. *marcelae*, this species bears several unique puzzling features not referable to hypogean life (especially the genital morphology) that hindered any straightforward assessment of its systematic affinities, even at a coarse familial level. The only few presumed similarities (though intriguing) were preliminarily found by comparison with Gonyleptidae Tricommatinae (as restricted by [23]), and with the monotypic genus *Osornogyndes* Maury, 1993, an alleged “Gonyleptidae Pachylinae” from Valdivian forests in southern Chile [24], *ca*. 600 km from Doña Otilia cave. The mentioned difficulties are magnified by the current systematic framework of Laniatores (and Gonyleptoidea), not stable as yet, but under active revision and thereby subject to frequent changes. In recent years, advances in Gonyleptoidea, based on either morphological or molecular data [23, 25–28], derived in a better understanding of internal lineages, as well as in the recognition of new or re-ranked families (e.g., Cryptogeobiidae, Gerdesiidae, Nomoclastidae, Metasarcidae) and some major, well-supported clades (e.g., Laminata, an unranked clade of Gonyleptoidea [25]). Noteworthily, further novelties are to be expected (A. Kury, in litt.). The challenging systematic assignment of *Otilioleptes* was then approached through cladistic and Bayesian phylogenetic analyses, to allow a closer comparison with relevant lineages of Gonyleptoidea. Results led to the conclusion that the new genus likely represents an early diverging lineage within Laminata, and that a new family, Otilioleptidae fam. nov., has to be established to account for this taxonomic singularity. Some evolutionary and paleoenvironmental scenarios are discussed, to speculate about the origins and survival of this awesome troglobite in such an inhospitable area.

## Material and methods

### Abbreviations

#### Morphology

Pp: pedipalp; Cx: coxa; VP: ventral plate of penis; vps: ventral process of stylus; dpg: dorsal process of glans.

#### Cladistic terminology

EW: equal weights; IW: implied weighting; k: concavity value for IW; Ci: consistency index; Ri: retention index, BS: Bremer support or decay index.

#### Bayesian terminology

Mk: Markov K-States model; Mkv: Mk version that conditions on variable characters; PSRF: Potential Scale Reduction Factor.

#### Family-group taxa

Go(-): Gonyleptoidea, narrow sense (excluding Stygnopsidae); TRIAE: Triaenonychidae; STGNOM: Stygnommatidae; POD: Podoctidae; ASS: Assamidae; EPE: Epedanidae; PYR: Pyramidopidae; STOPS: Stygnopsidae; AGO: Agoristenidae; STY: Stygnidae; CRYP: Cryptogeobiidae; GER: Gerdesiidae; NOM: Nomoclastidae; MET: Metasarcidae; COS: Cosmetidae; MAN: Manaosbiidae; CRA: Cranaidae; AMP: Ampycinae; GON: Gonyleptidae *s*.*s*. (in this study represented by Pachylinae, Gonyleptinae, Goniosomatinae); TRIC: Tricommatinae.

#### Unranked clades

L: Laminata; Mic: Microsetata; GG: Greater Gonyleptidae (= G-sl + MAN); G-sl: Gonyleptidae *sensu lato* (TRIC+CRA+AMP+GON); G-ss: Gonyleptidae *sensu stricto* (TRIC+AMP+GON); O+O: *Otilioleptes* + *Osornogyndes*; C+M: Cosmetidae + Metasarcidae; T+C: Tricommatinae + Cranaidae.

#### Repositories of material examined

CDA: Colección de Arácnidos, Cátedra de Diversidad Biológica II, FCEFyN, Universidad Nacional de Córdoba; FML: Fundación Miguel Lillo, San Miguel de Tucumán; LEA: Collection Luis E. Acosta, Córdoba (housed in CDA); MACN: Museo Argentino de Ciencias Naturales “Bernardino Rivadavia”, Buenos Aires; ZFMK: Zoologisches Forschungsmuseum Alexander Koenig, Bonn (material studied at MACN).

### Phylogenetic analysis

The systematic relationships of *Otilioleptes* gen. nov. were tackled through the incorporation of the new species in the phylogenetic hypothesis proposed by Kury & Villarreal [25] for Gonyleptoidea, henceforth referred to as ‘K&V’. This hypothesis is well-suited to this purpose since it includes adequate representation of major lineages in the superfamily and appropriate outgroups, it has many points of agreement with published molecular phylogenies [26, 27], and the character set is almost entirely applicable to scrutinize the new taxon. Moreover, 35 out of 77 characters (46%) used in K&V [25] refer to the penis morphology, what represents an extra bonus, given the peculiar genital morphology and the somatic simplicity of *Otilioleptes*. The original K&V matrix was enhanced with the addition of 12 terminals, aside from the new genus. *Osornogyndes tumifrons* (“Gonyleptidae”), *Tricommatus brasiliensis*, *Tricommatus giuponii* and *Caramaschia singularis* (Tricommatinae) were included to test their presumed affinities with *Otilioleptes* (see comments in the taxonomic section below). *Eusarcus hastatus*, *Eubalta meridionalis*, *Pachyloides hades*, *Acrographinotus* sp., *Acutisoma longipes* (Gonyleptidae), *Zalanodius convexus*, *Spinopilar moria* (Cryptogeobiidae), and *Maracandellus* sp. (Assamidae) were added to reinforce the representation of these families in the dataset. Because published data were not enough to complete the scoring for the new characters of eleven terminals, these were replaced by confamiliar substitute species, for which samples were available for observation. Conversely, the taxon list was simplified by the removal of one nomoclastid, two cranaids and three ampycines from the original matrix. The final list of 45 terminals is given in Table 1, along with a detail of sources used for scoring the character states.

**Table 1:** List of terminals in the cladistic and Bayesian analyses, with literature sources or voucher specimens used to complete the scoring of taxa and characters.

FAMILY: *Species—*Voucher—Literature source	K&V 2015 *
1. TRIAE: *Acumontia succinea* Mendes & Kury, 2012—[29]	=
2. STGNOM: *Stygnomma fuhrmanni* Roewer, 1914—[30]	=
3. POD: gen. sp.—Thailand: Naratiwal Prov., Waeng District, Hala Bala W.S., Research Station, N 5°47’44.8” E 101°50’4.2”, 190–200 m, 13-14.x.2003, ATOL Expedition 2003, 1 ♂, 1 ♀ (MACN)	Repl
4. ASS: *Maracandellus* sp.—Thailand: Chiang Mai Prov., Doi Inthanon N.P., nr. intersect. rd. to Mae Chaem and checkpoint, wet primary forest, N 18°31’33.2”; E 98°29’57.7”, ca. 1800 m, 3.x.2003, ATOL Expedition 2003, 1 ♂, 1 ♀ (MACN)	Repl
5. ASS: *Ayenea trimaculata* Santos & Prieto, 2010—Gabon: Ogoové-Ivindo, near Ntenkelé (0°31.4’N, 12°31.5’E), 550 m a.s.l.. 12-viii-2011 (B.A. & S.R. Huber), 2 ♂♂, 1 ♀ (ZFMK)	Add
6. EPE: *Metepedanulus* cf. *flaveolus—*Malaysia-Borneo: Niah Cave N.P., forest near cave (3.814°N, 113.771°E), 40 m a.s.l., 28.vii.2014 (B. A. Huber), 1 ♂ (ZFMK Op.00387)—Malaysia-Borneo: Sabah: Mt. Kinabalu, Poring hot springs, forest near the beginning of Kipungit Trail (6.048°N, 116.706°E), 450 m a.s.l., 7.viii.2014 (B.A. Huber, S.B. Huber), 1 ♀ (ZFMK Op.00384)	Repl
7. PYR: *Pyramidops pygmaeus* Loman, 1902—Sharma, P., unpubl. photos	=
8. STOPS: *Paramitraceras granulatum* Pickard-Cambridge, 1905—Cruz-López, J.A., unpubl. photos—[31]	=
9. AGO: *Globibunus rubrofemoratus* Roewer, 1912—[32]	=
10. STY: *Ricstygnus quineti* Kury, 2009—[33]	=
11. STY: *Stygnus mediocris* (Roewer, 1931)—Ecuador: Napo: Limoncocha, 7.iv.1984 (A. Roig), 1 ♂, 1 ♀ (MACN)—[33]	Repl
12. CRYP: *Zalanodius convexus* (Mello-Leitão, 1940)—[23]	Repl
13. CRYP: *Spinopilar moria* Kury & Pérez-González, 2008—[19]	Add
14. CRYP: *Cryptogeobius crassipes* Mello-Leitão, 1935—Brasil: Rio de Janeiro, Praia Vermelha, 20-xi-1990 (A. Kury), 1 ♂, 1 ♀ (LEA 000.294)	Add
15. GER: *Gerdesius mapinguari* Bragagnolo, Hara & Pinto-da-Rocha, 2015—Pinto-da-Rocha, R (unpubl. photos)—[28]	=
16. NOM: *Quindina albomarginis* (Chamberlin, 1925)—Panamá: Barro Colorado island, viii-1985 (G. Mora), 1 ♂, 1 ♀ (LEA 000.419)	=
17. NOM: *Nomoclastes quasimodo* Pinto-da-Rocha, 1997—Pérez González, A (unpubl. photos)—[33]	=
18. NOM: *Zamora* sp.—Ecuador: Prov. Napo: Cantón Quijos: Yanayacu Biological Station. Night collecting along trail. Nov. 26, 2009 (E. Tapia coll, Niarchos Expedition), 1 ♂, 1 ♀ (MACN)	Repl
19. MET: *Incasarcus dianae* Kury & Maury, 1998—[34]	=
20. MET: *Metasarcus* sp.—Argentina: Salta, Aguas Blancas, ii-1997 (J.L. Farina, M.D. Romero & P. Romero), 1 ♂ (LEA 000.420)—Argentina: Salta, Aguas Blancas (R.P. 19, km 7), 29-i- al 13-ii-2001 (J.L. Farina, M.D. Romero), 1 ♂, 2 ♀♀ (LEA 000.421)	Repl
21. MET: gen. sp.—Argentina: Tucumán: Apeadero Militar General Muñoz (subida a Tafí del Valle), 1620 m, 12-ii-1995 (L. Acosta, A. Peretti, M. Acosta), 1 ♂, 2 ♀♀ (LEA 000.117)	Repl
22. COS: *Cynorta conspersa* (Perty 1833)—[35]	=
23. COS: *Gryne orensis* (Sørensen, 1879)—Argentina: Formosa, Herradura, Camping La Florencia, 3-xii-2011 (J. Vergara, R. González-Ittig. L. Vaschetto), 5 ♂♂, 4 ♀♀, 2 juv. (CDA 000.879)	Repl
24. COS: *Gnidia holmbergii* (Sørensen, 1884)—Argentina: Salta: Termas, 4 km E Rosario de la Frontera, 19.ii.1995 (L. Acosta), 3 ♂♂, 3 ♀♀ (LEA 000.206)	Repl
25. MAN: *Syncranaus cribrum* Roewer, 1913—Pinto-da-Rocha, R (unpubl. photos)—[36]	=
26. MAN: *Saramacia lucasae* (Jim & Soares, 1991)—Pinto-da-Rocha, R (unpubl. photos)—[36]	=
27. CRA: *Chiriboga albituber* Roewer, 1959*—*Ecuador: Pcia. Pichincha, 10 km Oeste Nono, 4.v.1982 (A. Roig), 2 ♂♂, 2 ♀♀ (MACN)	=
28. CRA: *Zannicranaus monoclonius* Kury, 2012—[37]	=
29. CRA: *Phalangodus* sp.—[38]	=
30. AMP: *Licornus tama* Villarreal & Kury, 2012—[39]	=
31. AMP: *Hutamaia caramaschii* Soares & Soares, 1977	=
32. AMP: *Ampycus telifer* (Butler, 1873)—[40]	=
33. GON: *Discocyrtus testudineus* (Holmberg, 1876)—Argentina: Entre Ríos, Strobel, 24-iii-2006 (L. Acosta, M. García), 8 ♂♂, 19 ♀♀ (LEA 000.358)	Repl
34. GON: *Gonyleptes horridus* Kirby, 1818	=
35. GON: *Acanthopachylus aculeatus* (Kirby, 1818)—Uruguay: Cerro Arequita, 3-xii-1997 (L. Acosta), 3 ♂♂, 4 ♀♀ (LEA 000.150)	=
36. GON: *Pachyloides hades* Acosta, 1989—Argentina: Tucumán, El Infiernillo, 5-iv-1986 (L. Acosta), 1 ♂, 1 ♀ paratypes (CDA 000.014)—[41]	Add
37. GON: *Eusarcus hastatus* Sørensen, 1884—Argentina: Misiones, Comandante Andresito, 13-xii-2012 (L. Vaschetto, R. González Ittig, S. Poljak), 5 ♂♂, 1 ♀ (CDA 000.877)	Add
38. GON: *Eubalta meridionalis* (Sørensen, 1902)—Chile: Magallanes, Reserva Forestal Laguna Parrillar, 50 km SO de Punta Arenas, 28-29-i-1988 (E. Maury), 6 ♂♂, 6 ♀♀ (LEA 000.413)	Add
39. GON: *Acrographinotus* sp.—Perú: Ancash, Quebrada Ishinca, 18-vi-1998 (J.A. Ochoa C.), 5 ♂♂, 16 ♀♀, 2 juv. (LEA 000.025)	Add
40. GON: *Acutisoma longipes* Roewer, 1913—Brasil: São Paulo, São José dos Barreiros, Parque Nacional Serra da Bocaina, rio Mambucaba (1400 m), 21-24-iii-1997 (Pinto-da-Rocha, Campaner & Vanin col.), 3 spec. (LEA 000.292)—[42]	Add
41. TRIC: *Tricommatus brasiliensis* Roewer, 1912—[23, 43]	Add
42. TRIC: *Tricommatus giuponii* (Kury, 2003)—[44]	Add
43. TRIC: *Caramaschia singularis* Kury, 2002—[45]	Add
44. *Otilioleptes marcelae* gen. nov., sp. nov.—This paper	Add
45. *Osornogyndes tumifrons* Maury, 1993—Chile: Osorno, 3 km S of Maicolpué, Bahía Mansa, 21-xii-84–3-ii-85 (S. & J. Peck), 1 ♂ paratype (MACN 9117), 1 ♀ paratype (MACN 9118)—[24]	Add

* References for **‘**K&V 2015’ column: ‘ = ‘, a species included in the matrix of Kury & Villarreal [25]; ‘Repl’, a replacement terminal; ‘Add’, a new terminal, added to K&V [25] matrix.

Fifteen extra characters were incorporated. For characters 8, 40, 52, 53, 66 and 67, definitions were slightly modified, or additional states were added, to describe more accurately the new terminals, especially *Otilioleptes*. Seven characters of the original matrix were set aside, either because of difficulties in their scoring (23. Pedipalpal femur, ventrobasal cluster of setiferous tubercles; 48. Lateral borders of pars distalis; 62. Median field of scale-bristles, shape; 63. Median field of scale-bristles, structure; 64. Lateral fields of scale-bristles, shape, and 77. Tarsal aggregate pores) or because of redundancy with character 39 (76. Tarsal claws of legs III-IV, structure). To make comparisons easier, characters numbering of K&V [25] was maintained; columns for the unused characters 23, 48, 62, 63 and 64 are left blank in the matrix (S1 Table), but 76 and 77 were simply suppressed: the 15 new characters were then appended to the matrix, as characters 76–90. The matrix was edited using Mesquite version 2.75 (freely available at http://mesquiteproject.org), then exported as Nona file (.ss) for cladistic analysis and to Nexus format (.nex) for the Bayesian approach. The final list of characters and states (all non-additive) is detailed in S1 Text; the matrix of 45 terminals x 85 active characters is given in S1 Table.

#### Cladistic analysis

Tree search under parsimony was executed in the free software TNT version 1.1 (http://www.lillo.org.ar/phylogeny/) [46], with the “traditional search” strategy (1000 replicates, 5 random seed, branch swapping with SPR). Memory setting was raised to 10000 trees. Because of the high degree of homoplasy and to replicate methods used by K&V [25] as closely as possible, the parsimony analysis was made under implied weights (IW [47]), a method that assigns a higher weight to the characters having less homoplasy. IW analyses were performed using seven different concavity (k) values (1, 3, 6, 9, 12, 15, 18), together with a run based on equal weights (EW, non-weighted) for comparison. Lower values of k penalize more strictly the homoplastic characters; when values of k increase, the function becomes similar to the linear function of EW. Trees obtained with TNT were opened in Winclada 1.00.08 [48] to trace character changes (unambiguous optimization) and to obtain Ci and Ri. Branch support was assessed with TNT, by calculating three resampling-based measures: standard Bootstrap (sample with replacement), jackknifing (independent character removal, 36% removal probability) and symmetric resample (33% change probability). In all cases, resampling was applied to each concavity value, upon 500 replicates using traditional search, with frequency difference (GC) as output, and a cut-off = 1 (branches below this value are collapsed). Besides, Bremer support or “decay index”, i.e., the number of extra steps needed to collapse a given branch of a most parsimonious tree [49], was estimated with a script running in TNT. The search started with a 50-replicates parsimony ratchet, followed by 10 random addition sequences (TBR branch swapping) and saving up to 10 trees per replication. After that, 15 search cycles for suboptimal trees were performed, applying a stepwise increase of the suboptimal threshold and the tree buffer in each run (from “sub 5; hold 10000; sub 10; hold 20000; sub 20; hold 30000;…” up to “sub 90; hold 150000;”). In all command lines, swap was done until the tree-buffer was filled.

#### Bayesian methods

The matrix was also analyzed using an alternative phylogenetic approach, likelihood-based, the Bayesian inference, which has become widely accepted in molecular systematics [50, 51]. Most Bayesian methods for phylogenetic reconstruction were specifically designed to handle molecular data and can be adjusted to a wide range of evolutionary models. In contrast, a straightforward model, the so-called ‘Mk model’ (= Markov k-states model), as proposed by Lewis [50], is considered suited for analyzing discrete morphological data, which have very different properties to the molecular ones [51]. The suitability of Bayesian methods to analyze morphological data is currently on debate: some researchers asseverate that results obtained under the Mk model outperform those of parsimony [52, 53], while others advocate for exactly the opposite, considering the Mk model too unrealistic and inadequate for morphological data sets [54–56]. Without aiming to take part in the controversy, in this paper a Bayesian model was performed to compare with topologies obtained under parsimony. The Mk model was run using the free software MrBayes 3.2.7 (available at https://nbisweden.github.io/MrBayes/) [57], by executing a nexus (.nex) file that contains both the matrix (S1 Table) and the prompts to define parameters and perform the analysis. Following the Mk model postulates [50], rates of evolution were allowed to vary across sites by assuming a discrete gamma distribution, and the character acquisition bias was solved by excluding constant characters, allowing only variable characters in the data (parameters rates = gamma and coding = variable; this model frequently referred to as ‘Mkv’). *Acumontia succinea* was selected as the outgroup. Six independent MCMC (Markov chain Monte Carlo) were run simultaneously, each run consisting of six separate chains; searches were performed for 2x10^6^ generations (number of cycles for the MCMC algorithm), and sampled every 500 generations, with the first 25% of samples discarded as a burn-in (burninfrac = 0.25). MCMC diagnostics was calculated every 5000 generations (diagnfreq = 5000). The resulting consensus tree was displayed and edited with FigTree 1.4.3 (http://tree.bio.ed.ac.uk/software/figtree/).

### Taxonomic methods

This work did not involve sampling of specimens, but was based on material deposited in the Invertebrates Collection, Fundación Miguel Lillo (FML), San Miguel de Tucumán, a permanent public repository open to scientific research (Curator: M.A. Peralta, maperalta@csnat.unt.edu.ar). Specimens were examined, measured and drawn using a Leica Wild M3C stereomicroscope with camera lucida. Photographs of type specimens were kindly taken by Abel Pérez-González and Willians Porto using a Leica DFC 290 digital camera attached to a Leica M165C stereomicroscope; different focal planes of this image were combined using Helicon Focus Pro (www.heliconsoft.com). Descriptions follow [58], especially for the use of prolateral / retrolateral as a topological reference on appendages, and for the notation of the tarsal formula and pedipalp spination. In the latter, the use of square brackets is here proposed to denote contiguous spines sharing the same tegumentary elevation (e.g., iIii[**I**i] means, from basal to distal, a sequence “small-large-small-small-large-small spines”, the two latter arising from a bifid base); bold is used to indicate the largest spine in a group, if applicable. An acute cuticular projection is termed a ‘spine’ if articulated into a socket, or an ‘apophysis’ when emerging smoothly from the tegument [58]. All measurements are in mm. Relative lengths (ratios) for legs and pedipalps express *n*-times the scutum length; for the basal tarsomere, *n*-times the sub-basal tarsomere; in both cases, an “x : x : x : x” notation separates leg pairs. Male genitalia were studied and illustrated in temporary mounts in glycerol [58] using a Nikon E200 microscope with camera lucida. Macrosetal patterns on the VP were described following [25]. Line drawings were digitized using the free software Inkscape 0.92 (www.inkscape.org).

### Nomenclatural acts

The electronic edition of this article conforms to the requirements of the amended International Code of Zoological Nomenclature (https://www.iczn.org/the-code/), and hence the new names contained herein are available under that Code from this electronic edition. To fulfil the requirements for availability stated in Art. 8.5. of the Code, this published work and the nomenclatural acts it contains have been registered in ZooBank (Official Register of Zoological Nomenclature) (http://zoobank.org), and they accordingly have their respective LSID (Life Science Identifier). The electronic edition of this work was published in a journal with an ISSN, and has been archived and is available from the following digital repositories: PubMed Central and LOCKSS. ZooBank LSIDs can be resolved and the associated information viewed through any standard web browser by appending the LSID to the prefix “http://zoobank.org/”. The LSID for the present electronic publication is urn:lsid:zoobank.org:pub:CDE55C43-9233-48F4-A44F-A390E4AEC60E.

### Cartography

The location map was designed with the free, open-source geographic information system software QGIS 2.4.0—Chugiak (https://qgis.org/), using spatial data freely available at https://www.worldwildlife.org/publications/terrestrial-ecoregions-of-the-world (shapefiles based on [59]) and http://www.diva-gis.org/Data.

## Systematic results

### Cladistic analysis

#### Trees obtained with implied weights (IW)

**Fig 2:**
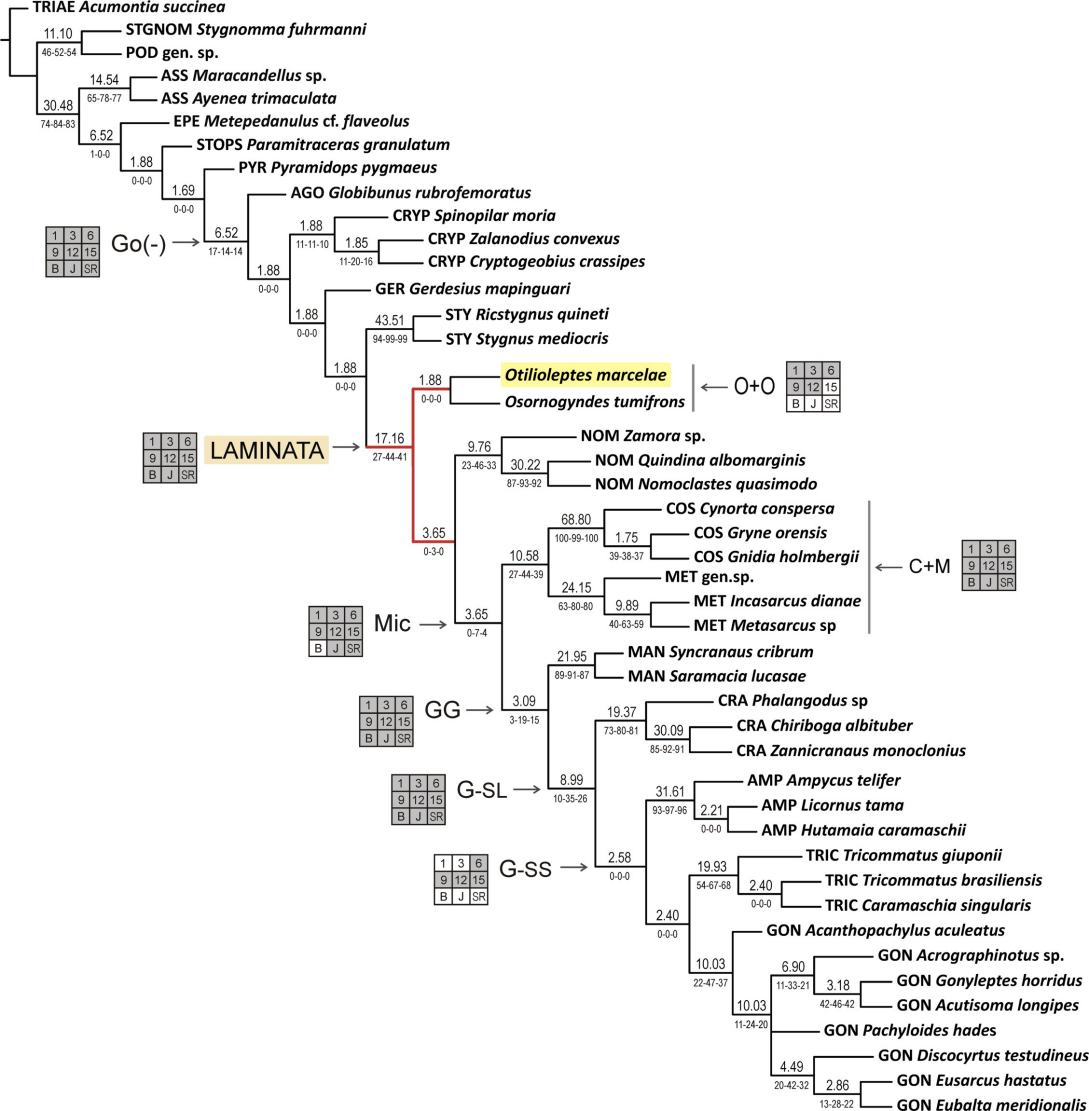
Cladistic relationships of Gonyleptoidea and placement of *Otiloleptes marcelae* gen. nov., sp. nov. Single most parsimonious tree obtained with implied weights (IW), k = 6 (Ci: 0.30, Ri: 0.59, tree length: 510 steps). Number above each branch indicates Bremer support; below, values of bootstrap, jackknife and symmetric resample (B-J-SR). Grid (‘Navajo rug’) beside a clade summarizes whether it is retrieved (grey cell) or not (white cell) in selected treatments (k = 1, 3, 6, 9, 12, 15, B, J, SR resamplings of the k = 6 tree). In red: sector of K&V [25] hypothesis where *Otilioleptes* is incorporated (basal in Laminata).

They displayed a quite similar topology across a wide concavity span (k = 1 to k = 13), and at the same time, replicated most branches of K&V hypothesis [25]; the single tree resulting with k = 6 was the preferred hypothesis (Fig 2). Three major unranked clades recognized in K&V [25], Laminata (L), Microsetata (Mic) and Greater Gonyleptidae (GG), were consistently retrieved with IW from k = 1 to k = 13 (S1 Fig), although only L had a relatively high BS value (Fig 2). On the contrary, the extent of Gonyleptoidea constitutes a remarkable disagreement between analyses. The monophyly of ‘Gonyleptoidea in a broad sense’ (i.e., including Stygnopsidae, as the sister clade of all the rest [25]), was not supported in this study. Instead, Gonyleptoidea was recovered in the “narrow sense” [26, 60], that is, restricted to Agoristenidae + the rest (STOPS excluded and displaced one node towards the root; the clade, with low BS, is then abbreviated ‘Go(-)’. Between k = 1 and k = 13, *Otilioleptes* and *Osornogyndes* grouped in a clade (O+O), as the sister group of the ‘Laminata’ in their original scope (S1 Fig; Table 2). Considering the defining apomorphies of the clade Laminata (recognition of a well-defined VP and associated features [25]; see also S2 Fig), together with results of resampling analyses (see below), I here propose L to embrace *Otilioleptes* and *Osornogyndes* as its most basal branches. At lower concavities (k = 1 to k = 5), Tricommatines grouped with Cranaidae (T+C, forming the sister clade of AMP+GON), but between k = 6 and k = 13, tricommatines became the sister group of Gonyleptidae. In both k intervals the clade “Gonyleptidae *sensu lato*” (G-sl) was recognized (TRIC+CRA+AMP+GON), while a subordinated cluster, “Gonyleptidae *sensu stricto*” (G-ss): (AMP (TRIC+GON)) was generated with k = 6–13 (Fig 2; S1 Fig; Table 2). The latter hypothesis would be in better agreement with the current assignment of Tricommatinae as a subfamily within Gonyleptidae; with T+C, to recognize TRIC as a subfamily would require considering CRA as a member of the family as well. Concavities k = 14 and k = 15 resulted in the pectination of *Osornogyndes* and *Otilioleptes* (in this order) in the base of NOM (S1 Fig). From k = 16 onwards (IW was tested up to k = 50), *Otilioleptes* and *Osornogyndes* were placed more internally than NOM, pectinated at the base of Microsetata (roughly resembling the topology obtained with EW, see below). In any case, no concavity supported any close relationship between Tricommatinae and *Otilioleptes*.

**Table 2:** Recovery of the relevant clades, monitored in different analytical treatments.

		Go(-)	L	O+O	O+O +NOM	Mic	C+M	GG	G-sl	T+C	G-ss
IW—concavity	k 1	+	+	+	–	+	+	+	+	+	–
	k 3	+	+	+	–	+	+	+	+	+	–
	k 6	+	+	+	–	+	+	+	+	–	+
	k 9	+	+	+	–	+	+	+	+	–	+
	k 12	+	+	+	–	+	+	+	+	–	+
	k 15	+	+	–	+ ^OT^	+	+	+	+	–	+
	k 18	–	+	–	–	+	+	+	+	–	+
resample k = 3	B	17	32	– *	–	–	26	–	6	20	–
	J	17	54	– ^OT-L^	–	–	42	20	37	39	–
	SR	14	49	– ^OT-L^	–	–	37	13	25	37	–
resample k = 6	B	17	27	– *	–	–	27	3	10	12	–
	J	14	44	– ^OT-L^	–	7	44	19	35	17	–
	SR	14	41	– ^OT-L^	–	4	39	15	26	18	–
resample k = 15	B	16	10	– *	–	–	25	1	6	–	2
	J	10	26	– *	–	5	44	4	10	–	12
	SR	13	18	– *	–	3	38	5	10	–	11
EW	EW-str	–	–	– ^OS^	–	+	+	–	–	–	+
	EW-50	–	–	– ^OS^	–	+	+	–	–	–	+
resample EW	B	11	2	– *	–	–	18	–	–	–	1
	J	5	3	– *	–	–	21	–	–	–	4
	SR	7	3	– *	–	–	22	–	–	–	3
Mkv		+	+	–	–	–	+	–	–	–	+

**Treatments:** Implied weighting (IW) with seven different concavity values (k); equal weights (EW), strict consensus (EW-str) and majority rule (EW-50); resampling with bootstrap (B), jackknife (J) and symmetric resample (SR): values indicate branch support when ≥1; Mkv: results obtained with the Bayesian Mkv analysis.

**Clades:** Go(-): Gonyleptoidea, narrow extent (without STOPS); L: Laminata; O+O: *Otilioleptes* + *Osornogyndes*; O+O+NOM: (*Otilioleptes* (*Osornogyndes* (Nomoclastidae))); Mic: Microsetata; C+M: Cosmetidae + Metasarcidae; GG: Greater Gonyleptidae; G-sl: Gonyleptidae *sensu lato*; T+C: Tricommatidae + Cranaidae; G-ss: Gonyleptidae *sensu stricto*. If O+O is not recovered, it is indicated whether *Otilioleptes* is more basal than *Osornogyndes* (^OT^, denoted as ^OT-L^ if the most basal of all Laminata), *Osornogyndes* is more basal (^OS^), or these terminals collapse in a basal polytomy of Laminata (*).

#### Trees obtained with equal weights (EW)

This analysis yielded 33 equally parsimonious trees. The consensus of EW trees (either strict or by majority rule) failed to recover most of the branches referred to above (S1 Fig). Of the major clades recognized by K&V [25], only Microsetata and C+M (Cosmetidae + Metasarcidae) were retrieved (the latter is indeed the only clade present in all and any treatments). Very relevant lineages, like Go(-), L or GG did not appear using EW. Even worse, in some cases (Cryptogeobiidae, Nomoclastidae), familiar clusters lost their cohesion and got dismantled. Alike with k ≥ 16, *Osornogyndes* and *Otilioleptes* resulted basal to Microsetata using EW, leaving NOM more basally in Laminata; however, in this case, the intrusion of Stygnidae amongst NOM actually dissolved the monophyly of the latter and the Laminata under EW. Although trees obtained with EW were shorter (length 503) than with IW (Table 3), they show many topological inconsistencies, especially when resampling methods are considered.

**Table 3:** Summary results of the eight analyses (k = 1, 3, 6, 8, 12, 15, 18; equal weights) performed on the matrix of S1 Table.

Concavity (k)	Trees retained	Tree length	Ci	Ri	Best score (fit)
1	1	515	0.30	0.58	49.46307
3	1	515	0.30	0.58	36.75061
6	1	510	0.30	0.59	27.30825
9	1	510	0.30	0.59	21.88885
12	1	510	0.30	0.59	18.32948
15	3	507	0.31	0.59	15.78042
18	3	505	0.31	0.59	13.85395
None (EW)	33	503	0.31	0.60	n/a

#### Resampling methods

Bootstrap, jackknifing and symmetrical resampling were performed for three concavities (k = 3, k = 6, k = 15), to represent their respective intervals. In general, Go(-), L and C+M were the best-supported clades with all three methods (Table 2). In contrast, Mi, which was retrieved across all tested concavities, received weak or no support at all when resampled. Similarly, the clade O+O, consistently formed from k = 1 to k = 13, disappeared in all resamplings; depending on the *k* used, these terminals either formed a pectination basal to all Laminata (*Otilioleptes* at the base, followed by *Osornogyndes*), or both genera separately collapsed into a basal polytomy of that large clade (S1 Fig; Table 2). In no case, *Otilioleptes* or *Osornogyndes* were placed outside Laminata, or shifted into a more terminal position, or moved far away from each other. Resampling of EW trees resulted in an unexpected resurgence of the main clades Go(-) and L, missing in the regular analysis, although always with low support (Table 2).

#### Bayesian analysis

The six independent runs of the Mk model converged on very similar posterior estimates, with an average standard deviation of split frequencies of 0.009148. PSRF (a convergence diagnostics that compares the estimated between-chain variance with the within-chain variance for a parameter) was close to 1.0 both for parameters and for trees, indicating that the sample from the posterior probability is good. The analysis did not yield a fully resolved tree, resembling results obtained with EW. Branches that were weakly supported in the precedent parsimony analyses collapsed here in several polytomic nodes (S3 Fig). Some major clades identified in most variants of the cladistic analysis were recognized with Bayesian methods as well (Table 2), in most cases having a high posterior probability (S3 Fig). These include: Go(-), i.e., the Gonyleptoidea without STOPS; Laminata, as one of the best supported major internal clades within Go(-); Gonyleptidae *sensu stricto* (G-ss), and the clade comprising Cosmetidae and Metasarcidae (C+M); these results give thus independent evidence in favor to the strength of those parts of the phylogenetic hypothesis. Like with EW, CRYP is not retrieved as monophyletic, and all three terminals of this family separately joined the Go(-) polytomy (S3 Fig). The Bayesian analysis did not resolve the exact placement of *Otilioleptes* and *Osornogyndes*, which do not form a clade O+O, but individually take part of the large Laminata polytomy that comprises other five well-supported lineages: NOM, C+M, MAN, CRA and G-ss.

#### Position of *Otilioleptes*

Although not an aim of this study, the cladistic analysis served as a kind of proof of the robustness of K&V hypothesis [25]–and it passed the test quite well. Results with IW maintained most major clades, despite the inclusion of several new taxa (chiefly the Tricommatinae, *Osornogyndes* and two extra Cryptogeobiidae), the replacement of others, and the incorporation of additional characters. As seen, the only main disagreement was the extent of Gonyleptoidea, here recognized in the “narrow sense” because of the exclusion of STOPS. It should be noted that clades resolving these relationships have relatively low BS (Fig 2) and that the inclusion of this family (or not) within Gonyleptoidea is at least contentious in the literature (see e.g., [25, 26, 60–62]).

On the other hand, this analysis reinforced one previously suspected relationship: the otherwise isolated *Otilioleptes* is consistently placed near the bizarre ‘pachyline’ *Osornogyndes*. Both genera are likely the basal members of the Laminata (S1 Fig), although in a few treatments they may appear related to NOM (IW k = 14–15) or shifted one further step inside the tree (EW). In any case, support for a clade O+O is weak; in the selected tree (Fig 2, see also S2 Fig) this clade has a very low BS (1.88) and is defined by a single homoplastic feature, #53 (state 0 > state 1: presence of vps, also shared with clades GON, CRYP and one single TRIC) , so that a sister-taxa relationship of these two genera does not appear stable enough. The recovery of O+O with some IW concavities (k = 1–13) is, indeed, the only evidence to group both genera, say, in the same family. However, evidence against this supposed confamiliar status appears stronger: O+O is not retrieved from k = 14 onwards, neither with EW nor any resampling method (S1 Fig). This conclusion is endorsed by relevant alpha-taxonomic differences too, as detailed under the generic description. The Bayesian consensus tree, much less resolved than parsimony, did not support a clade O+O either, not even the topological vicinity of *Otilioleptes* and *Osornogyndes*, except for both belonging to the Laminata (S3 Fig). Grouped in a clade or not, the recurrent topological proximity of these genera (S1 Fig), can be explained by their shared basal condition, not necessarily for being sister-taxa. From these results it is clear that *Osornogyndes* can no longer be maintained in Gonyleptidae Pachylinae, nor assigned to Otilioleptidae fam. nov. or any other existing suprageneric clade. *Osornogyndes* is therefore kept as “Family uncertain” within Gonyleptoidea Laminata until its relationships are investigated in more depth.

Characters optimized in the *Otilioleptes* branch (S2 Fig) include three non-homoplasious autapomorphies, two of which refer to genitalia: #66 (state 4 > state 7: macrosetae A-B transverse) and #81 (state 2 > state 4: vps truncate), the remaining one to exomorphology: #82 (state 1 > state 0: frontal hump equal-sized as ocular mound). Homoplasious apomorphies in this clade comprise: #6 (0 > 1, presence of frontal hump, shared with GER, two MET and G-ss); #21 (0 > 1, medial subapical spine on pedipalp femur, with several back and forths, and independent appearances in *Cryptogeobius*, *Quindina*, MET, MAN and GON); #40 (1 > 0, tarsal process, separately lost in *Otilioleptes*, MET and TRIC); #76 (1 > 0, multiple macrosetae AB, shared with *Phalangodus* sp. and *Acrographinotus* sp.); #78 (0 > 2, macrosetae A-B displaced to the truncus, present in *Acrographinotus* sp.); #79 (1 > 0, macrosetae C shifted proximad, a state scattered among *Nomoclastes*, ASS, STY and STOPS); #80 (1 > 0, apical border of VP cleft, as in *Gonyleptes* + *Acutisoma*, MET gen. sp. and *Zamora*); #85 (1 > 2, pedipalp coxa moderately elongated) and #88 (1 > 2, coxa III long, like GER, *Zalanodius* and *Ayenea*). Results proved that Tricommatinae are not related to *Otilioleptes*, as initially believed, but they are close to Gonyleptidae and allies instead (Fig 2).

## Taxonomic treatment

### Family Otilioleptidae fam. nov

urn:lsid:zoobank.org:act:7973595B-826D-4F82-8AE3-95BF3CFC6E2C

**Type genus.** *Otilioleptes* gen. nov. Family monotypic.

**Diagnosis.** Opiliones, Laniatores, Gonyleptoidea, Laminata. Small, long-legged harvestmen, of delicate habitus, thoroughly unarmed. Ocular mound as a blunt granulous mound, with extremely rudimentary eyes. Scutum almost smooth, with five mesotergal areas, sulci almost vanishing. Free tergites I-III and dorsal anal plate unarmed. Stigmatic segment broad, posterior border sub-straight, surpassing the coxa-trochanter IV joint. All appendages elongated. Chelicera and pedipalps with normal appearance; pedipalp femur armed with a strong medial subapical spine; tibia and tarsus with proventral and retroventral rows of long spines. Legs I-IV unarmed in both sexes. Coxa II longer than III on ventral view. Distal end of coxa IV not entirely fused to the stigmatic segment. Coxa IV unarmed or with a short, conic prolateral apophysis in male (extremely reduced in female), and a small retrolateral one (both sexes). Distitarsi tri-segmented in all legs. Tarsal process on legs III-IV absent. Tarsal formula: 6 : 9–11 : 6–7 : 6–7; basal tarsomere elongated, about 2–3 times as long as the preceding one on leg I, three times or more on legs II-IV. Sexual dimorphism negligible, limited to subtle size differences of the prolateral apophysis on coxa IV, and legs slightly longer in males. Penis slender and straight. VP devoid of ventral microsetae cover, its apical portion forms a translucent spatula-like platform. Macrosetae groups as typical for Laminata: distal group (macrosetae C), basal group (A+B), and small D and E macrosetae. All setae noteworthily displaced proximad, with group A+B of multiple strong transverse setae, inserted on the truncus end. Glans aligned with truncus, without dpg; stylus oblique, cylindrical, bearing a simple blunt vps.

### Genus *Otilioleptes* gen. nov.

urn:lsid:zoobank.org:act:458D142C-74B5-4FA8-A142-DB20EF1D768E

**Type species.** *Otilioleptes marcelae* sp. nov. here designated. Genus monotypic.

**Etymology.** The generic name merges the word *Otilia* (after Cueva Doña Otilia, the type locality), with final vowel changed to -*o* for euphony, and the ending–*leptes* (from Greek: *leptos*,meaning thin, fine, delicate), as used in several gonyleptid genera (e.g., *Gonyleptes* Kirby, 1818). Grammatical gender is masculine.

**Distribution.** Only known from the species type locality (Cueva Doña Otilia, Payunia region, Mendoza Province, Argentina).

**Diagnosis.** Gonyleptoidea, Laminata, Otilioleptidae fam. nov. The generic diagnosis is to be referred to the family diagnosis above.

**Affinities.** A mix of several presumably primitive characters, together with the uniqueness of the genital shape, strongly suggests that *Otilioleptes* belongs to an isolated gonyleptoid lineage. The presence of a well-defined ventral plate (VP) and a simple, unfolded glans support the placement of the new genus amidst Gonyleptoidea families grouped by K&V [25] in their unranked clade Laminata. This position is consistently recovered in all phylogenetic analyses, in most cases *Otilioleptes* occupying a basal topology, in the vicinity of the Chilean “gonyleptid” genus *Osornogyndes*.

**Fig 3:**
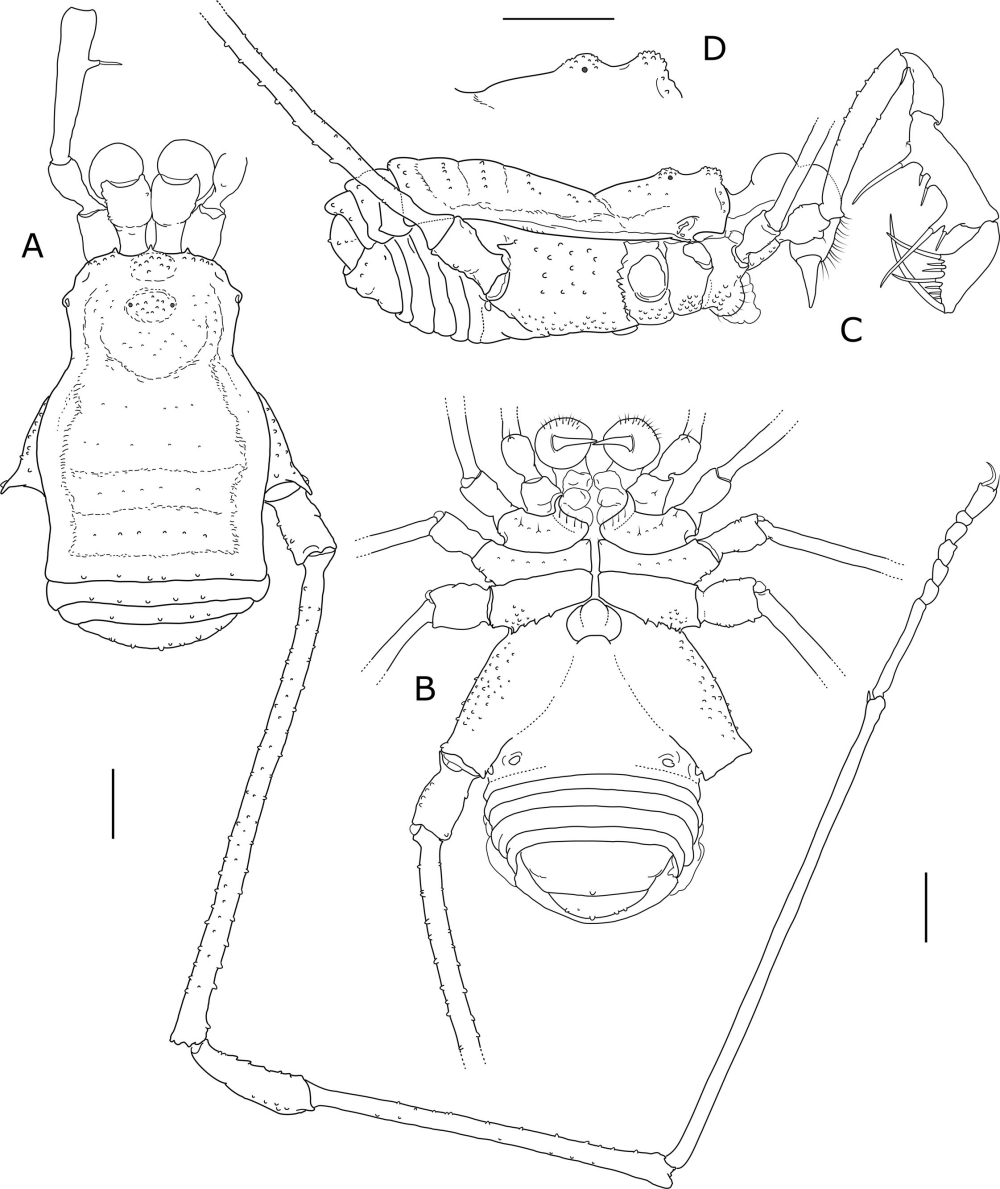
*Otilioleptes marcelae* gen. nov., sp. nov., holotype male (FML-OPIL 00211), habitus. A: Dorsal view. B: Ventral view. C: Lateral view. D: Detail of ocular mound and frontal hump, lateral view. Scale bars: 1 mm.

**Alpha-taxonomic remarks.** The vps-bearing stylus of *Otilioleptes* has some faint resemblance with Gonyleptidae. Other features, however, would be clearly atypical for that family, even if troglomorphic characters are set aside: (1) the almost lack of sexual dimorphism, (2) the coxa IV not completely fused to the stigmatic area, and (3) the caudal border of the latter (sternite III) broad and with posterior margin sub-straight, not deeply concave as in most gonyleptids. At the same time, character (3) might represent a similarity of *Otilioleptes* with Tricommatinae, currently considered a small subfamily within Gonyleptidae [23, 25]. Also, tricommatines do not have a tarsal process on tarsi III-IV (absent in the new genus too, but present in most gonyleptids). Tricommatines were not included in K&V phylogeny [25], but these observations made them prime candidates to be added in the cladistic analysis. Stylus and vps of *Otilioleptes* have some apparent resemblance with *Osornogyndes*. The rest of the penis, however, looks very dissimilar: shape in *Osornogyndes* is ‘normal’, not elongated, and macrosetae are arranged in a different way (see below). *Osornogyndes* is unusual in several external features too, some of them recalling *Otilioleptes*, like the lack of armature in scutum, legs and ocular mound, and the almost complete absence of sexual dimorphism; moreover, the stigmatic segment is broad as well [24]. Despite those coincidences these genera differ in many relevant aspects, like the general habitus (*Otilioleptes* is more slender, with much longer appendages), the scutum outline, and the design of mesotergal areas (in *Osornogyndes* the first sulcus is straight; in *Otilioleptes* this sulcus is curved, as if the diffuse area I were divided) (cf. Figs 1 and 3). The shape of chelicerae, pedipalps and coxa IV, as well as the tarsal formula, are very distinctive too.

### *Otilioleptes marcelae* sp. nov.

urn:lsid:zoobank.org:act:B805C6B2-6BCB-4243-B5F4-927332C84231

**Type series.** Holotype male and 1 female paratype (FML-OPIL 00211), Argentina, Mendoza Province: Cueva Doña Otilia, Malargüe, ~350 m from entrance, 20 August 2006 (A. Salvo & M. Peralta); 1 male paratype (FML-OPIL 00218), same loc., 04 April 2012 (M. Peralta).

**Type locality.** Cueva Doña Otilia, near Malargüe, Mendoza Province, Argentina; *ca*. 35°57' S 69°42' W.

**Etymology.** The specific name is dedicated to the speleologist Marcela Peralta, in recognition to her determination in unveiling the biospeleological value of Cueva Doña Otilia, as to provide sound evidence for the urgent need to protect this beautiful and fragile ecosystem.

### Description

General color uniform whitish-yellowish, completely depigmented and with hyaline appearance (Fig 1). Detailed measurements of holotype male, male and female paratypes: Table 4.

**Table 4:** Measurements (mm) of the holotype male, and female and male paratypes of *Otilioleptes marcelae* gen. nov., sp. nov.

	HOLOTYPE ♂ FML 211	PARATYPE ♀ FML 211	PARATYPE ♂ FML 218
Body length	5.20	5.69	4.89
Scutum, length	**4.38**	**4.26**	**4.00**
maximal width	3.03	2.92	2.86
Prosoma lenght	1.61	1.55	1.50
width (at ozopores)	2.30	2.19	2.06
width at constriction	2.17	2.00	1.85
Leg I, total length	**14.56**	**12.85**	**13.07**
trochanter	0.65	0.56	0.58
femur	3.46	3.14	3.25
patella	1.23	1.12	1.09
tibia	2.58	2.21	2.24
metatarsus	3.95	3.39	3.55
tarsus (total)	2.69	2.43	2.36
Basal tarsomere	0.98	0.84	0.86
Leg II, total length	**27.20**	**23.56**	**24.19**
trochanter	0.72	0.62	0.65
femur	6.77	5.85	5.91
patella	1.61	1.46	1.38
tibia	5.11	4.45	4.52
metatarsus	6.00	5.23	5.54
tarsus (total)	6.99	5.95	6.19
Basal tarsomere	2.26	1.87	2.05
Leg III, total length	**18.85**	**16.49**	**16.80**
trochanter	0.74	0.64	0.67
femur	5.35	4.68	4.92
patella	1.53	1.40	1.35
tibia	3.40	2.97	3.03
metatarsus	5.11	4.41	4.58
tarsus (total)	2.71	2.39	2.24
Basal tarsomere	1.14	1.00	1.03
Leg VI, total length	**24.68**	**21.75**	**22.19**
trochanter	1.01	0.80	0.85
femur	7.08	6.15	6.34
patella	1.85	1.66	1.65
tibia	4.98	4.49	4.53
metatarsus	6.58	5.72	6.06
tarsus (total)	3.18	2.92	2.77
Basal tarsomere	1.34	1.10	1.22
Pedipalp, total length	**9.14**	**8.62**	**8.48**
trochanter	0.74	0.60	0.67
femur	2.53	2.34	2.32
patella	1.09	1.01	0.97
tibia	1.73	1.70	1.66
tarsus	1.56	1.45	1.40
claw	1.49	1.51	1.46
Chelicera, distal part length	1.92	1.80	1.79
basichelicerite length	0.77	1.10	1.18

**Dorsal scutum** (Fig 3A). Outline type α [25]; abdominal scutum only a little wider than prosoma. Scutal narrowing feeble; from there, lateral sides of prosoma diverge anteriorly, rendering this tagma slightly sub-trapezoidal. Prosoma width is maximal at the ozopores, which are conspicuous, oval, and bordered by a shoulder-like tegumentary projection each. Frontal hump (Fig 3D) is a blunt protuberance on the anterior border, covered by coarse granulation (the most ornate part on dorsal view, indeed). Similar granulation forms a row both sides on the anterior border. Ocular mound low and unarmed, covered by small granules; eyes are hard to find among granulation since corneas are extremely reduced (in all specimens, two spots of retinal pigment deepened, probably because of retraction of internal tissues during fixation). Scattered small granules behind and beside the ocular mound give this part a tenuous rugulose look. Abdominal scutum little globose in lateral view, contrasting with the gently upwards sloping prosoma (Fig 3C). Scutal grooves almost faded away. Only in lateral view (and using side illumination), five faint scutal areas are insinuated; a row of minute granules each on areas III and IV help to identify them. Limit between lateral areas and mesotergum also feeble, denoted by an irregular ramp, which continues into the prosoma; extremely weak transverse sulci separating prosoma from opisthosoma, and mesotergum from area V. Scutum unarmed and smooth (matt), with finely granular texture on mesotergum; area V has 6–8 minute conic granules, sparsely aligned on its border. Free tergites unarmed, with similar granule rows as area V. Dorsal anal plate unarmed, with two horizontal rows of small granules.

**Venter** (Fig 3B). Ventral side of coxae covered by faint granulation; on coxa I, 3–4 setigerous granules on a row, plus many short setae along the sclerotized border of coxapophysis I; one row of five smaller setae on coxa II, plus one single, notorious one on coxapophysis II; even smaller setae on coxa III, one row with six, plus 2–3 additional setae posteriorly. Coxae I and II of similar length; apical end of coxa II diagonal, it surpasses moderately coxa III length. Prolateral border of coxa I with one granule, retrolateral border smooth; on coxa II, one prolateral and two retrolateral granules. Coxa I-II joint as a smooth sulcus; coxa II-III joint fixed, without articular serration; coxa III-IV joint with interlocking articular denticles. Distal end of coxa IV not fused to the stigmatic segment (separation arises more anteriorly than the stigma position itself). Stigmatic segment extended caudally beyond the coxa-trochanter joint, leaving the oval-transverse stigma distanced from the border; posterior edge of stigmatic segment sub-straight to faintly concave medially. Free sternites with rows of sparse tiny granules; ventral anal operculum with a few scattered grains.

**Chelicerae.** Basichelicerite elongated, especially the pedicel-like proximal portion; bulla distinct though low, unarmed except for a few sparse grains on the sides. Hand normal, conspicuously setose on its front surface, mostly near the finger joint.

**Fig 4:**
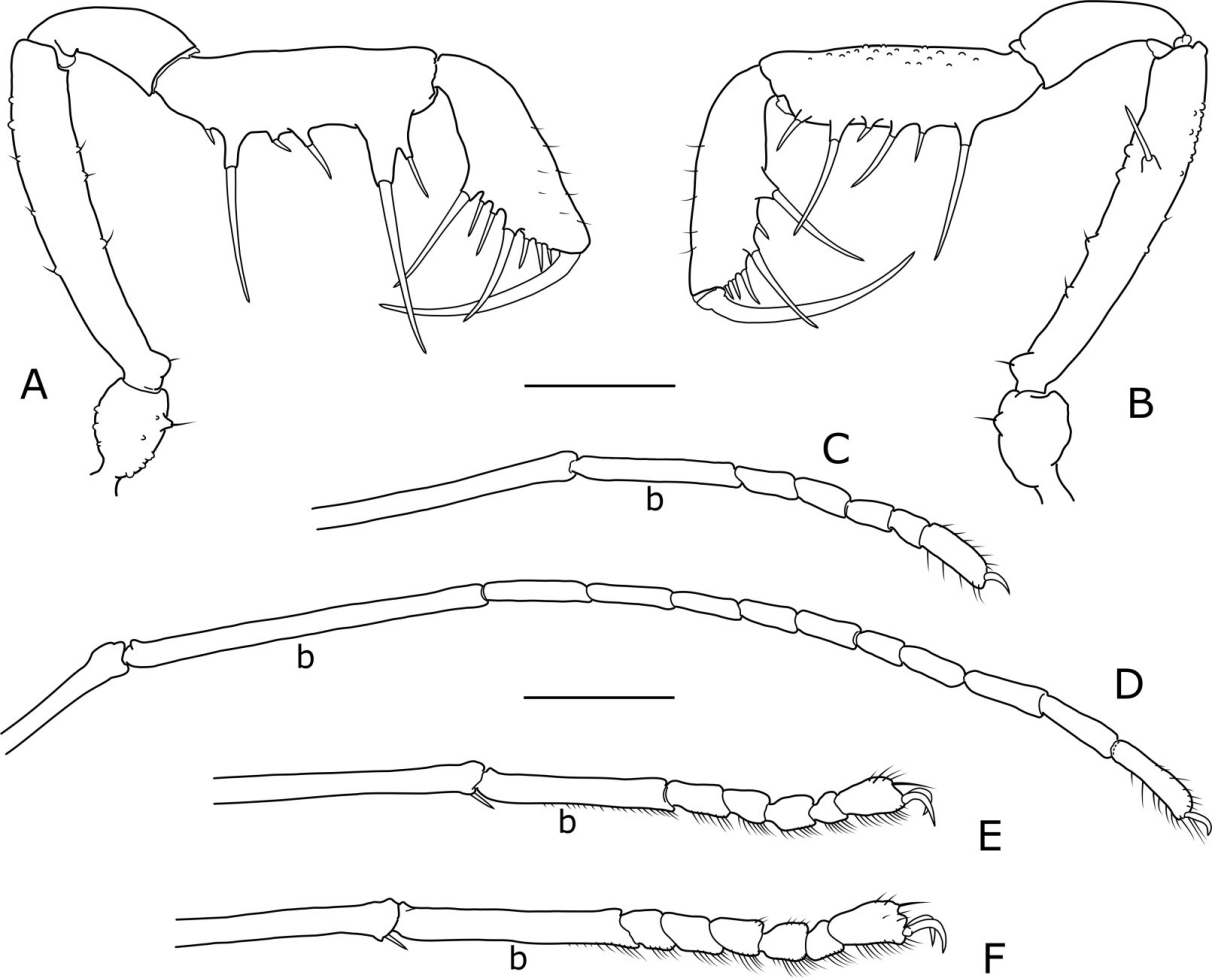
*Otilioleptes marcelae* gen. nov., sp. nov., holotype male (FML-OPIL 00211), appendages. A-B, right pedipalp, A: retrolateral view; B: prolateral view. C-F: right tarsi of legs, retrolateral view (basal tarsomeres indicated as “b”), C. Tarsus I; D. Tarsus II; E. Tarsus III; F. Tarsus IV. Scale bars: 1 mm.

**Pedipalps** (Fig 4A and 4B). All segments elongated; Pp length / scutum length ratio: 2.1 (males), 2.0 (female). Pp coxa elongated, it surpasses coxa I. Distal part of trochanter with scattered blunt grains and a small but conspicuous ventral setigerous tubercle. Femur long and slender, almost smooth except for a dorsal and a ventral row of vestigial granules, bearing delicate bristles; a basal, ventral setigerous tubercle, mirroring that of trochanter; and a large subapical medial spine on a raised socket between the distal and middle thirds, together with a small tubercle more apically. Patella elongated, smooth. Tibia smooth, armed with iIii[**I**i] ventrolateral and Ii**I**iIi ventromedial spines; ventral side flat, borne with sparse small spine-like bristles. Tarsus smooth, with scattered dorsal and lateral minute setae, and **I**iiIii ventrolateral, **I**iIii ventromedial spines. Spines on tibia and tarsus are long, spanning widely to the sides if seen from above. Claw as long as tarsus.

**Legs.** Slender and delicate, unarmed, with all segments elongated. Leg length / scutum length ratios: 3.3 : 6.0–6.2 : 4.2–4.3 : 5.5–5.6 (males), 3.0 : 5.5 : 3.9 : 5.1 (female). Dorsal border of coxae I-II with two blunt proximal tubercles each, associated with the ozopore complex. Trochanters I-III have some blunt conic grains on retrolateral and ventral sides. Femora, patellae and tibiae I-III straight and simple, just with rows of minute acute granules. Leg IV (Fig 3A). Coxa IV elongated, not globose; lateral sides granulous. Small, acute proapical apophysis; in the male, this apophysis is simple and unciform, curved over the coxa-trochanter joint; in the female, coxa IV has the same outline, but the apophysis takes the shape of a blunt grain. Tiny retroapical apophysis as a lobular projection in the space separating coxa and stigmatic segment. Trochanter long, covered by sparse grains, only a retrolateral pair and one retroapical a little larger. Femur, patella and tibia simple, with regular rows of conic granules. Tarsal segments: 6/6:9/11:6/6:7/7 (holotype ♂), 6/6:9/9:6/6:7/6 (paratype ♂), 6/6:9/9:7/7:7/7 (paratype ♀); distitarsus tri-segmented in all legs. Basal tarsomere elongated (Fig 4C–4F); ratio basal / sub-basal tarsomeres: 2.6–2.8 : 2.8–3.5 : 3.1–3.6 : 3.4–4.7 (males), 2.1 : 4.1 : 4.9 : 3.5 (female). Claws smooth. Tarsal process on tarsi III-IV absent, its position is occupied by an apical hair. Scopula present on tarsi III-IV, denser on the 4–5 distal tarsomeres.

**Fig 5:**
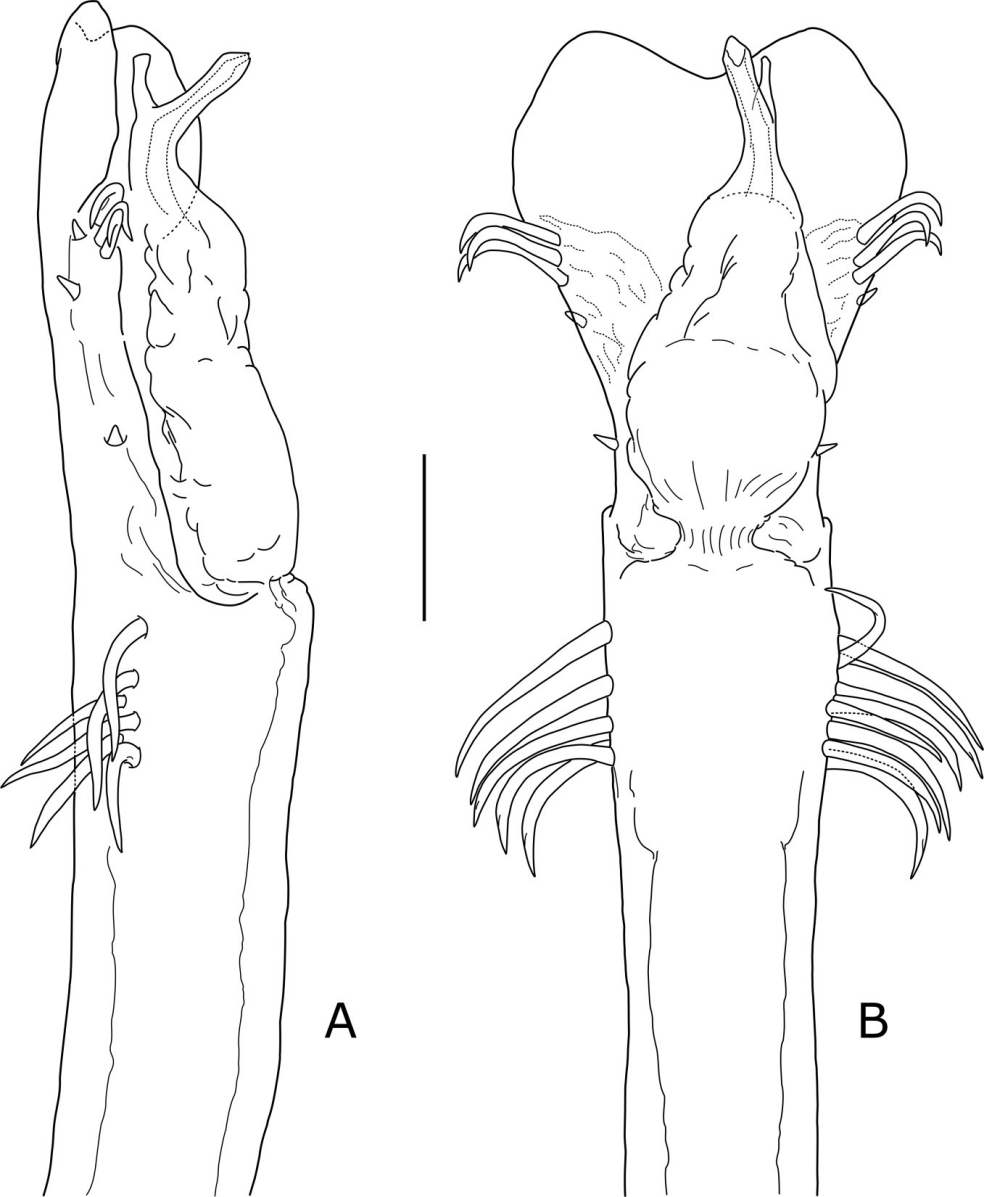
*Otilioleptes marcelae* gen. nov., sp. nov., holotype male (FML-OPIL 00211), distal end of penis. A. Lateral view (slightly rotated dorsad to reveal the vps), B. Dorsal view. Scale bar: 0.1 mm.

**Penis** (Fig 5A and 5B). General aspect elongated, very slender and straight, distally just a little arched ventrad; VP and glans aligned with trunk, without noticeable flexures. From the apical macrosetae anteriad, VP is expanded, remarkably thin and translucent, overall spatulate or petal-shaped; this flattened apical portion has lateral lobate borders, and distal edge concave. Distal group (macrosetae C) strongly displaced basally, arising on subdistal one-quarter of VP; it consists of 2–3 spine-like setae, long and apically curved, with two pairs of reduced macrosetae E, more ventrally. Middle group (macrosetae D) represented by an isolated, short seta (D1) on the VP narrowing. Basal group (A+B) strongly displaced beyond the glans–trunk boundary; it consists of 6–8 strong, long setae, whose sockets arrange longitudinally on the trunk sides; setae point almost straight to the laterals, giving the appearance of a mighty transverse armature. Ventral side of VP smooth, not covered by spiny mats. Glans–trunk articulation as a slight flexure; glans elongated and simple, just a little flattened and expanded sideways; there is no dpg. Stylus oblique, smooth, with no spination; it bears a simple, blunt vps arising in the point where the stylus changes its orientation from longitudinal to oblique.

### Genus *Osornogyndes* Maury, 1993

urn:lsid:zoobank.org:act:E394E57A-3AE1-4E53-881F-6242D8821FC7

*Osornogyndes* Maury, 1993: 100 [24]; Kury, 2003: 180 [63].

**Type species.** *Osornogyndes tumifrons* Maury, 1993, by original designation. Genus monotypic.

**Distribution.** Chile, Región de Los Lagos: Osorno Province.

**Fig 6:**
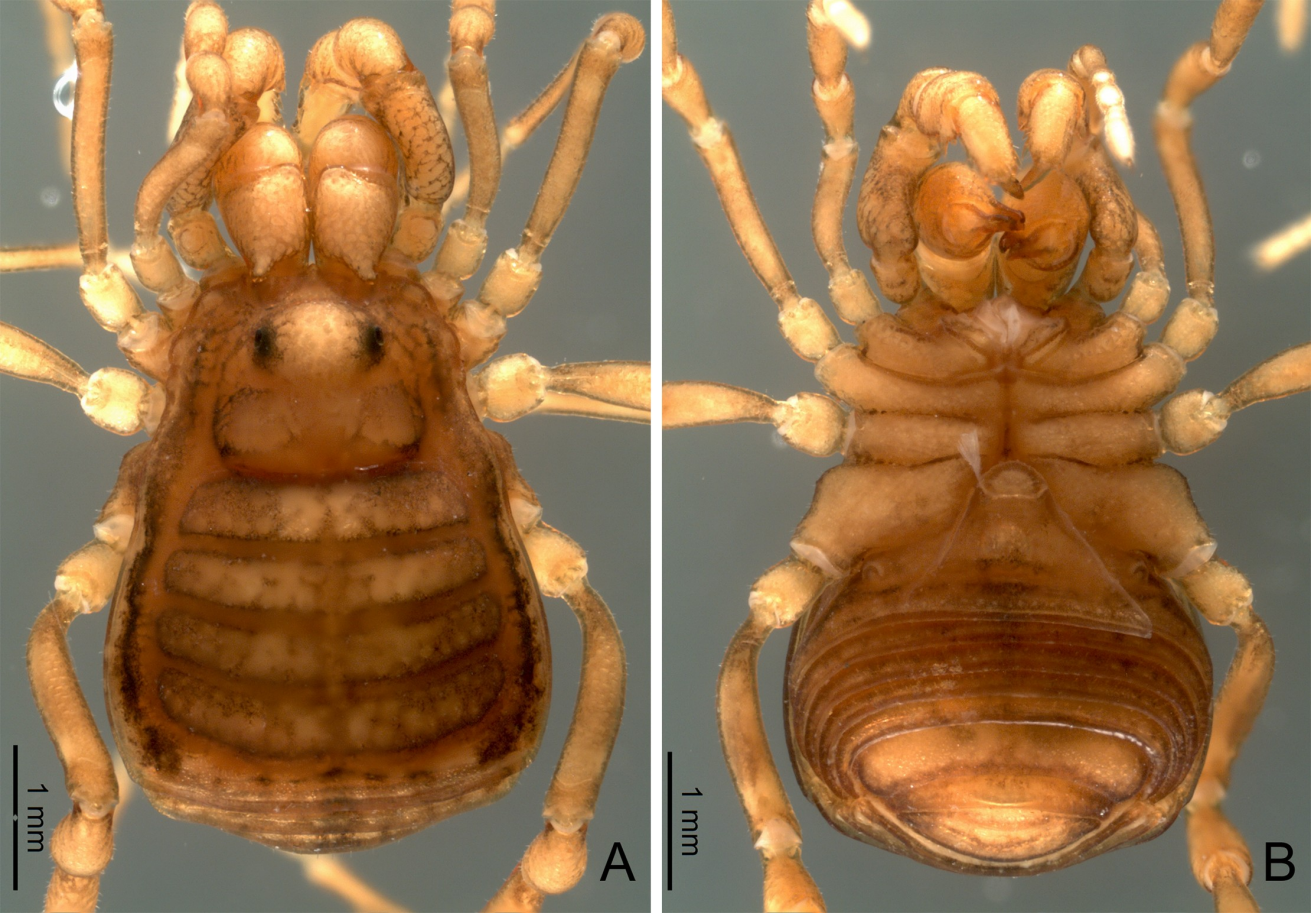
*Osornogyndes tumifrons* Maury, 1993, paratype male (MACN 9117), habitus. A. Dorsal view; B. Ventral view. Photos: Willians Porto.

**Fig 7:**
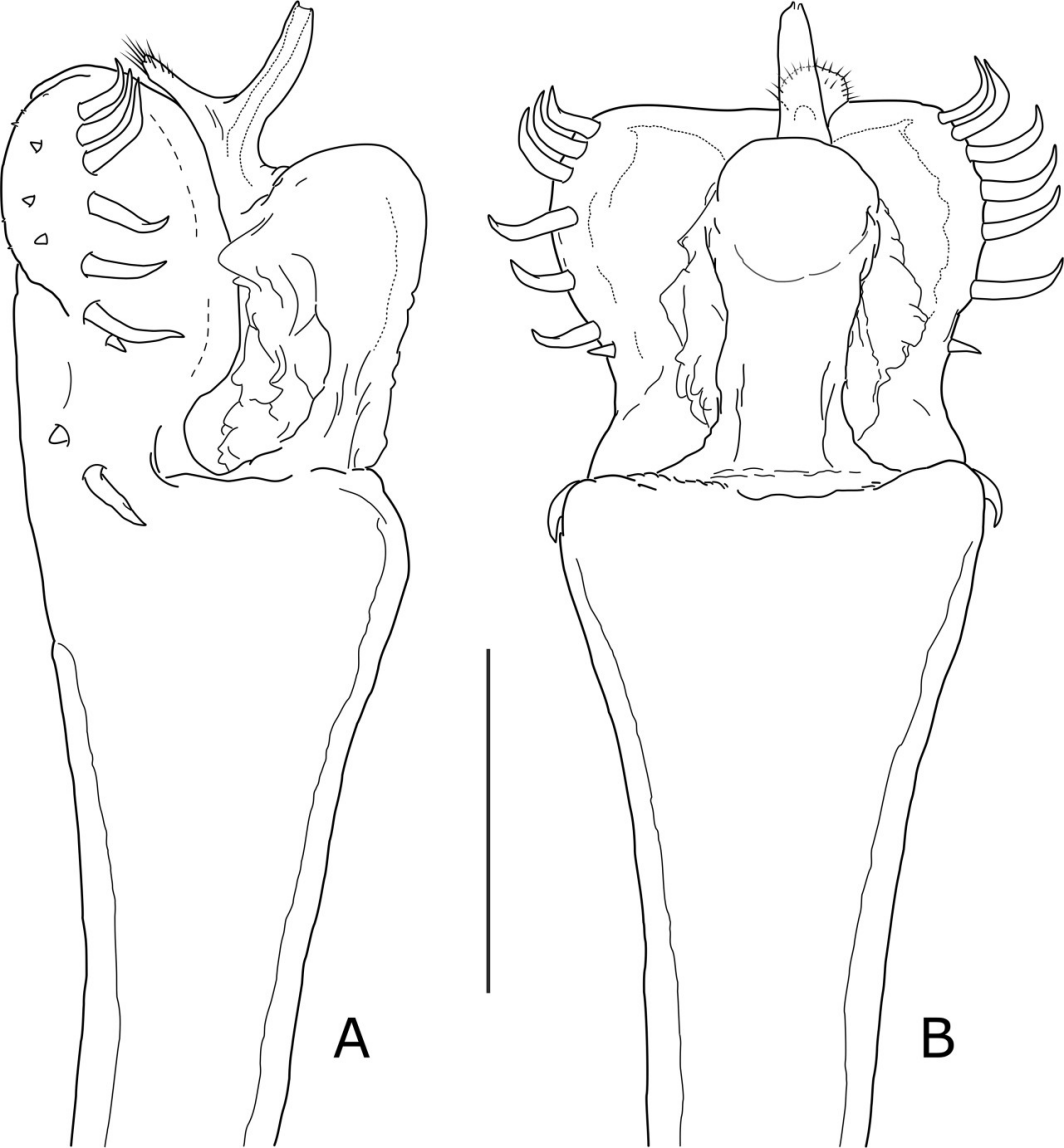
*Osornogyndes tumifrons* Maury, 1993, paratype male (MACN 9117), distal end of penis. A. Lateral view; B. Dorsal view. Scale bar: 0.1 mm.

**Diagnosis update.** Opiliones, Laniatores, Gonyleptoidea, Laminata, family uncertain (removed from Gonyleptidae Pachylinae, as hitherto assigned). Outline of dorsal scutum type θ, unarmed (Fig 6A). Front margin of prosoma without frontal hump, with three denticles. Ocular mound blunt, unarmed. Areas I-V well-defined by transverse grooves, area I entire (not divided in two halves by a longitudinal groove). Lateral areas, area V, free tergites, dorsal and ventral anal plates and sternites with a few granules. Border of stigmatic segment gently concave (Fig 6B). Cheliceral bulla armed with a conspicuous dorso-caudal apophysis in both sexes. Pedipalps: femur short, thicker than legs femora, without a subapical mesal spine; tibia and tarsus with a weak armature, the former having two or three pairs of ventral pairs of setigerous tubercles. All legs unarmed in both sexes, subequal and relatively short. Coxa II with apical end curved (Fig 6B), slightly longer than coxa III. Coxa IV short (not hypertelic), its distal end not completely fused to the stigmatic area. Tarsal formula: 4:5:6:6; distitarsi I-II tri-segmented. Tarsal process on legs III-IV vestigial, with a rigid seta. Sexual dimorphism represented by only subtle differences: in males, chelicerae and pedipalps are slightly more robust than in females, and the basitarsite I is a little swollen. Penis (Fig 7A and 7B) of a typical Laminata shape, with a well-defined VP (not flattened). Six pairs of equally sized marginal macrosetae along the distal half; the anterior three pairs are slightly distinct and can be interpreted as the C group; the rest may be a part of the basal group (macrosetae A), shifted distally; a short acute macroseta D near the caudal-most seta in that row (at the narrowing of VP). Lateral at the VP–truncus boundary, the remaining large macroseta A (which was overlooked in the original descriptions and figures [24]), together with a stump-like macroseta B. Small, tubercle-like setae subapical on the ventral side representing group E. Glans with a protruding dorsal convexity, without dpg. Stylus tubular, gonyleptid-like, with a simple, straight vps, apically peltate and covered by a spiny tuft (stylus was originally described as ‘divided into two similarly-sized branches’, and vps drawn as truncate [24]).

**Affinities.** Like *Otilioleptes*, *Osornogyndes* consistently occupies a basal position within the clade Laminata (Fig 2; S1 Fig). As stated above, these genera share many plesiomorphies, but evidence for considering them close relatives is weak. Otherwise, *Osornogyndes* is quite isolated. The cladistic analysis clearly supports its exclusion from Gonyleptidae.

### *Osornogyndes tumifrons* Maury, 1993

urn:lsid:zoobank.org:act:AC4EBC47-B01B-400E-8CD4-24D070A53C8F

*Osornogyndes tumifrons* Maury, 1993: 100, Figs 1–14 [24]; Kury, 2003: 180 [63].

**Type locality.** Sierras S of Maicolpué, Osorno Province, Región de Los Lagos, Chile; *ca*. 40°36'43"S 73°44'50"W.

**Fig 8:**
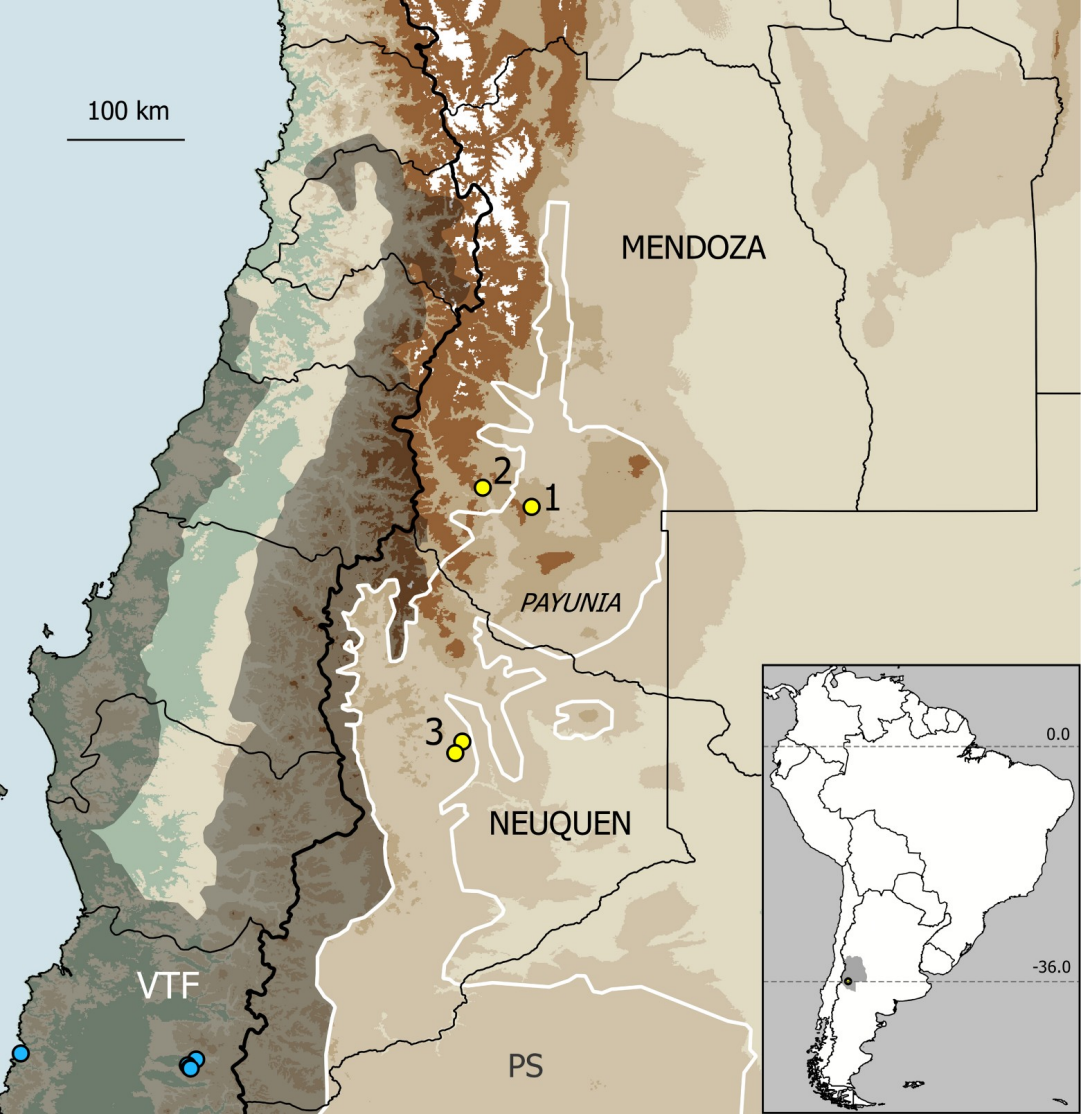
Location of Doña Otilia cave in the Payunia region (Mendoza Province, Argentina). The site is indicated as (1). Other caves in the area with records of Gonyleptidae: (2) Caverna de Las Brujas; (3) Caverna de Chorriaca and Caverna Aguada de la Mula, Neuquén Province. Light-blue dots: records of *Osornogyndes tumifrons* Maury, 1993 in Osorno Province, Chile. Ecoregions (following [59]): Patagonian Steppe (PS, white outline) and Valdivian Temperate Forests (VTF, shaded). Inset: position of Mendoza Province (grey) and the type locality (dot) in South America.

**Distribution.** This species was hitherto collected in two separate areas in Osorno Province, Chile (Fig 8): near the Pacific coast, south of Bahía Mansa (S of Maicolpué, type locality), and in the National Park Puyehue, close to the international boundary with Argentina (Anticura, Termas de Puyehue, Aguas Calientes, Los Derrumbes). The two areas belong to the Valdivian temperate forests ecoregion, with a record gap of about 190 km in between. Specimens were collected in leaf litter and under fallen logs [24].

**Material examined.** CHILE: Osorno, 3 km S of Maicolpué, Bahía Mansa, 21-xii-84–3-ii-85 (S. & J. Peck), MACN 9117, 1 ♂ paratype, MACN 9118, 1 ♀ paratype.

## Description of Doña Otilia cave

### Geological background

Doña Otilia is located at 1932 m a.s.l., not far from the karstic Caverna de Las Brujas. Both caves are placed in a geologically complex region, where the Andean ranges (to which Las Brujas belong) converge with the Payunia, an extensive back-arc volcanic field in southern Mendoza (Fig 8). The whole massif contains more than 800 monogenetic basaltic cones, together with a few polygenetic volcanoes fed by shallow magmatic chambers [64]; in many cases, eruptions were inferred to be of fissural type. According to the divisions of the Payunia, based on geographical position, age and geochemical characteristics [65, 66], Doña Otilia belongs to the relatively young ‘Llancanelo volcanic field’, where most volcanoes are arranged along short ENE–WSW trending fractures. The cave is placed near the margins of a large lava flow (16 x 4 km), with a thickness estimated in 8–12 m (E. Llambías, pers. comm. to C. Benedetto). This flow is made of olivinic basalt and consists of pahoehoe lava (a flow type prone to develop lava tubes [2, 22]). The Payunia is younger than 5 Ma, with an increased volcanic activity since 2 Ma [67]. No precise dating is hitherto available for Doña Otilia, but two hydromagmatic volcanoes of the Llancanelo basin (Malacara and Carapacho, placed at ~21 km and ~27 km from the cave) were estimated to be formed between 0.45 and 1 Mya [64] (that is, around Middle Pleistocene). Even younger dates were obtained at Cerro Jarilloso (a hydromagmatic volcano, at ~35 km; 0.16±0.07 Mya) and Cerro Las Ovejas (a scoria cone, at ~28 km; 0.28±0.02 Mya) [65]. Vulcanism appears to have persisted in the Holocene in a few sites around the Payún Matru volcano, 50 km south of Doña Otilia. In the Payunia, Pliocene-Holocene basalt layers (5.1 Mya and younger) lay over an older volcanic basement, dated in the Miocene [64].

### Epigean environment

**Fig 9:**
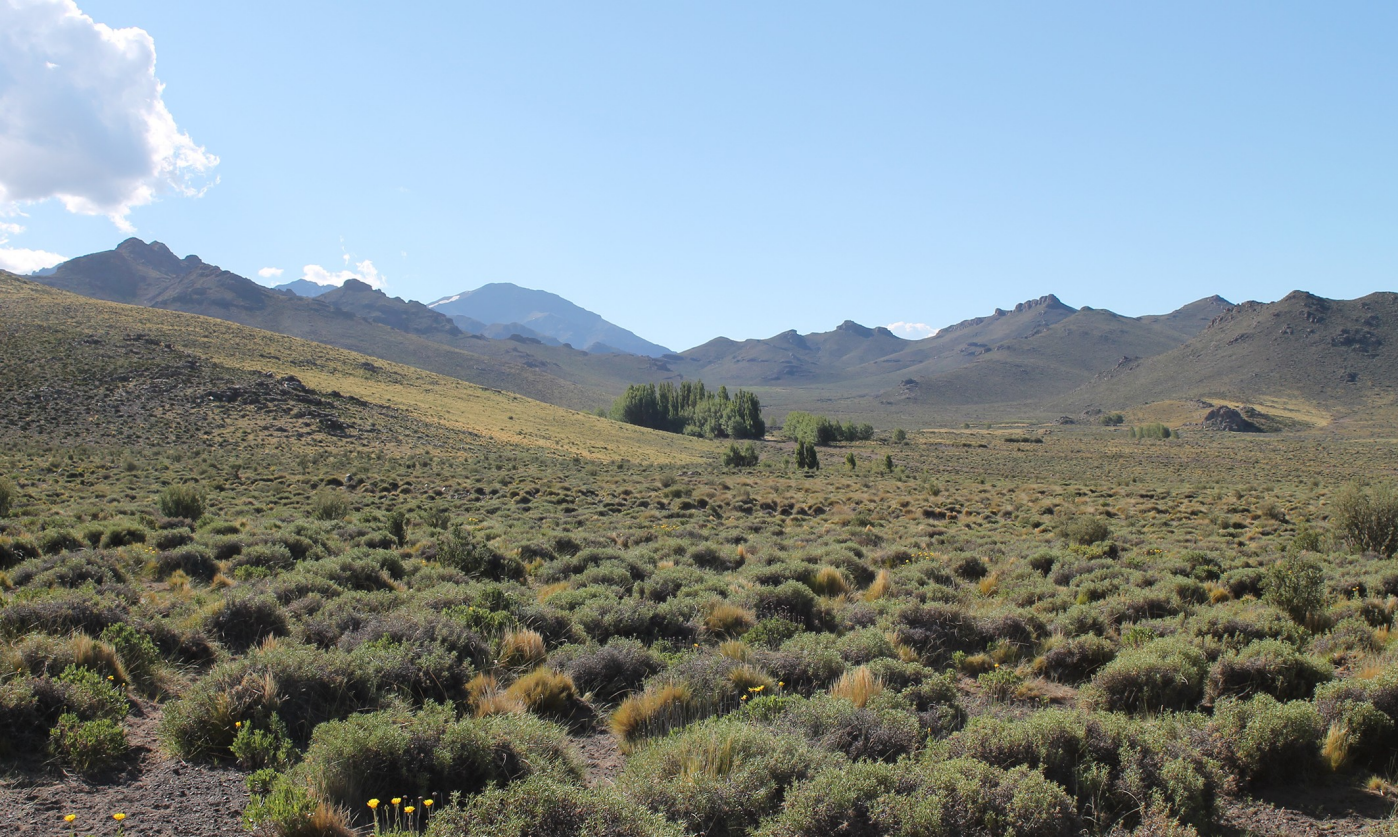
Epigean landscape in the Payunia, around Doña Otilia cave. Plant cover shows the characteristic pulvinate shrubs on sandy soil; the Sierras de Palauco are on the background.

This area is characterized by general aridity and typical north-Patagonian plant physiognomy (Fig 9). Climate is cold (annual mean 9–13°C) and dry, with frequent frosts [68], corresponding to BW (desert) in Koeppen´s system; annual rain in the Payunia averages 250–300 mm, and evapotranspiration rates are high [69]. Scarcity of precipitation is accentuated by its inopportune availability (winter), which results in little benefit for plants [70]. The floristic district of the Payunia is an intricate mosaic of arbustive steppes, in which local dominance varies according to substratum, elevation and topography [71]. Two xeric vegetal communities are the most characteristic, both with low and scarce plant cover [68]: grasslands of “coirón” (*Stipa* spp.) on deep sandy soils, and arbustive steppes on scoria plains, accompanied by pulvinate plants and sparse xerophytic shrubs (Fig 9). The district stretches northwards as a wedge between the Andean and the Monte ecoregions (Fig 8) so that some elements of the latter (e.g., *Larrea* spp.) are frequent [68, 71]. It is worth noting that the Payunia harbors a remarkable number of endemic plants, like the shrubs *Prosopis castellanosii* Burkart (Fabaceae), *Condalia megacarpa* A. Cast. (Rhamnaceae) and *Schinus roigii* Ruiz Leal & Cabrera (Anacardiaceae), as well as the herbaceous *Argylia robusta* Sandwith (Bignoniaceae) and *Pappostipa malalhuensis* (F.A. Roig) Romasch. (Poaceae) [70].

### Hypogean environment

**Fig 10:**
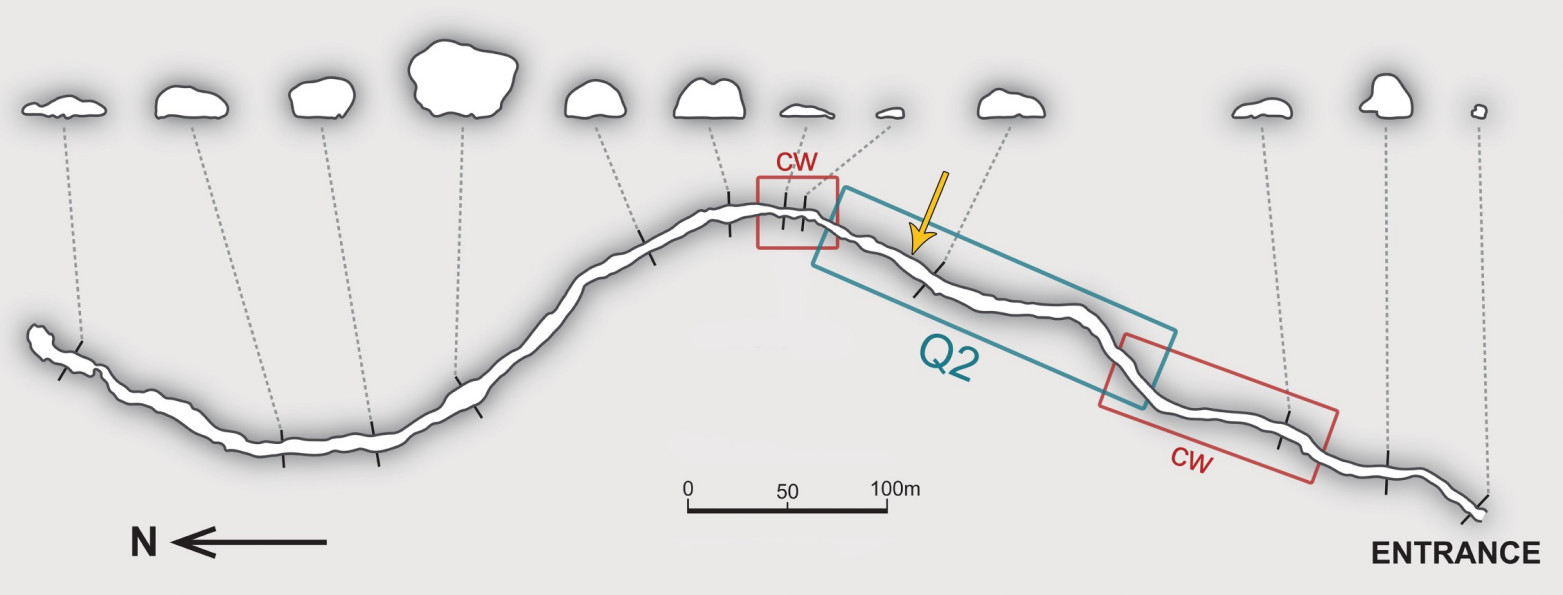
Plan view map of Doña Otilia cave. Transverse sections of the passage are displayed at the same scale. Q2: the humid sector; cw: crawlway passages. Arrow indicates the approximate location of Fig 11A and the collecting site of *Otilioleptes marcelae*. Redrawn from a 1973 survey made by C.A.E. (Centro Argentino de Espeleología).

The Payunia harbors several lava tubes that attracted increasing interest of speleologists, of which Doña Otilia cave is the longest [13]. This cave has only one small entrance (0.5 x 1 m), concealed in a shallow hole and almost unnoticeable in the outer landscape. This narrow slit gives way to a brief inclined descent covered by medium-sized boulders, referable to the ‘transition zone’ of the cave (*sensu* [22]), with virtually no twilight zone. The rest of the lava tube is a single S-N oriented gallery, 838 m long (Fig 10). As typical for volcanic caves, it describes a rather superficial and nearly horizontal trajectory (with a very gentle descending slope; maximum depth at 8 m beneath the surface). Sand, detritus, sparse gravels and fine sediment fill the bottom and give the effective passage a rough half-moon section in most of its length. Heights along the cave are varied: in the first half they range from ~1 m (a lengthy crawlway at the start) to 2.5 m, but in some deeper parts the conduit is higher than 6 m.

**Fig 11:**
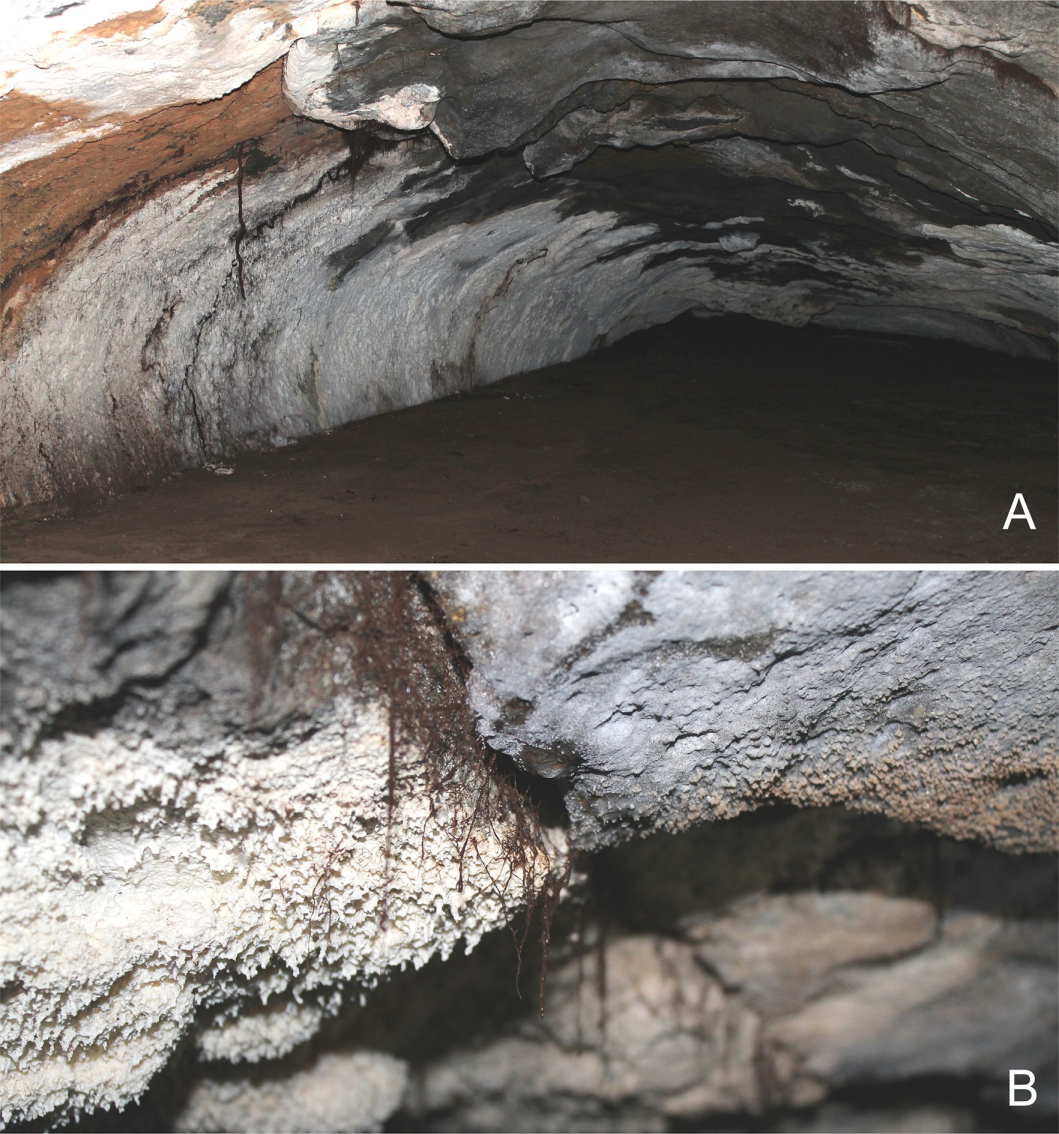
Hypogean environment in Doña Otilia cave. A: General view in the 2Q section of the cave (at about 350 m from the entrance); large roots can be observed hanging on the left side. B: Detail of speleothems and roots of epigean plants emerging through fissures on the ceiling and the walls.

The finely-grained floor helps maintain humidity in constant high levels, of around 80% [14], in contrast to the xeric epigean conditions. Temperatures (August) recorded in nine stations, from 8.5 m to 410 m from the entrance (7°-8°-10°-9°-9°-10°-10°-11°-11°C), evidenced stabilization in deeper sites (Instituto Argentino de Investigaciones Espeleológicas, unpublished, 2006). There are plenty of fissures and crevices in the ceiling and the walls that enable continuous groundwater filtration. The second quarter of the cave (henceforth referred to as ‘2Q’) has a distinctive character. It is a spacious sector (walking-sized, heights of 2–2.5 m) extending approximately between 230 m and 400 m from the entrance. There, water percolation is more intense than in other parts of the cave, favoring the formation of delicate gypsum and calcium carbonate speleothems over large surfaces in the ceiling and the walls (Fig 11A and 11B); this feature distinguishes Doña Otilia from other lava tubes in the area [13]. The 2Q sector is also peculiar in having many roots of epigean plants breaking through crevices, most of them small, tuft-shaped and aligned along the fissures (Fig 11B). Larger roots emerge in a few sites, either freely dangling or fit along irregular wall cracks, and may extend downwards to reach the humid floor. Both the roots and the speleothems are moistened by continuous water dripping; they are also very fragile so that any gentle rubbing makes them easily break off. In Hawaiian lava tubes, dangling tree roots were considered to constitute their primary energy source by supplying food, either directly as living or decaying roots, or by forming pathways for the infiltration of organically rich water [22]. Specimens of *Otilioleptes marcelae* were found in the 2Q sector of the cave, around 350 m from the entrance (Fig 10). They were discovered on the walls, either slowly crawling on gypsum-coated surfaces, or resting among the whitish speleothems, 30 cm from the floor, then manually-collected. Depigmentation makes these harvestmen hard to be detected on such a clear background (M. Peralta, in litt.). A few additional specimens were sighted in the same 2Q sector, hidden in deep fissures at 1.80 m or higher. Preliminary observations determined that hypogean conditions in Doña Otilia are suited to sustain a varied cave life [14, 72], although the presence of true troglobites in this lava tube has never been demonstrated before. The availability of organic material in a humid environment is distinctive for Doña Otilia among other basaltic caves in the Payunia. In addition to the mentioned percolating water and roots, seasonal and occasional inputs of surface water through the entrance, which drag organic rests of allochthonous origin (vegetal detritus, bone remains) into the cave, have been also described [14]. Surveys made in 1999, 2002 and 2006 revealed an invertebrate fauna of mites, collembolans, pseudoscorpions and earthworms in association with the roots, and myriapods, diplopods and Blattaria on the walls; spider exuviae and dead tipulids were detected in mesocavernous spaces (0.1–20 cm) in the ceiling [14]. Cadaveric entomofauna (dipterans and coleopterans, either troglophile or trogloxene) associated to scattered vertebrate remains (rodents, lizards) were also recorded (M. Peralta, in litt.). Except for a chilopod identified by L.A. Pereira as *Cryptops* sp. (Scolopendromorpha: Cryptopidae; FML-MYRIAP 00626), most of these samples remain undetermined and still await expert analysis.

### Conservation status

In the lack of any specific study on the conservation of Doña Otilia, only some general comments can be given here. Because of aridity, human population in the Payunia is limited to very low densities (0.05 inhabitants / km^2^). Consequently, human activity is minimal and with little impact on the area: up to the 19^th^ century it consisted of nomadic, hunter-gatherer aboriginal inhabitants, then replaced by a few, scattered rural settlements (‘puestos’), dedicated to extensive but low-scale livestock farming (especially goats), as seen today [73]. Oil extraction and mining activities were developed in some sites. More recently, the fascinating geography of the Payunia has motivated an increasing development of the ecotourism [73], including the so-called ‘speleotourism’ or recreational caving, which, if done without supervision or regulations, may represent a threat to fragile hypogean ecosystems. It appears uncertain that Doña Otilia can tolerate even moderate visitor traffic (speleothems get easily destroyed by unintentional rubbing with helmets, especially in narrow passages) so that this cave can be considered at least ‘vulnerable’; therefore, strict preservation measures would be highly desirable. However, Doña Otilia cave is placed in a private property, without any management strategy, other than the strict and efficient watch of Mr Martín Zagal, a rural resident in a neighboring ‘puesto’. Unfortunately, the cave is not embraced within the limits of any of two nearby provincial protected areas in the Payunia (http://www.areasnaturales.mendoza.gov.ar/): the Laguna Llancanelo Reserve (880 km^2^, aimed to conserve an outstanding wetland), and La Payunia Reserve (6 657 km^2^, comprising the Payún Matru and neighboring volcanic fields). Both reserves, together with large adjacent areas (encompassing Doña Otilia cave too) are comprised in the proposed ‘La Payunia, Campos Volcánicos Llancanelo and Payún Matrú’ unit (11 943 km^2^) [73], which, since 2011, is included in the Tentative Lists to integrate the UNESCO World Heritage (https://whc.unesco.org/en/tentativelists/5615/).

### An overview on cave-dwelling gonyleptoids

There are several cavernicolous Gonyleptoidea known from South American caves, in most cases reported from Brazil. The best-studied ones are indeed either troglophiles (species equally able to complete their life cycles both inside and outside the cave) or trogloxenes (those using caves as shelter, but which are regularly active outside the hole, for example to mate or forage), e.g., [74–77]. Among them are the various species of Goniosomatinae (Gonyleptidae) that seek for diurnal shelter in the cave, but walk outside to forage every night; the cave is surrounded by humid forests, what enables their daily epigean activity without risk [74, 76, 77]. Lack or scarcity of detailed biological knowledge often makes an ecological classification of cave harvestmen tentative, since true troglobites without marked troglomorphism are not rare, especially in tropical regions ([78]; A. Pérez-González, in. litt.), fading the morphological limits between troglobites and troglophiles. This uncertainty might be the case of *Eusarcus cavernicola* Hara & Pinto-da-Rocha 2010 [79], described from several caves in central Brazil (Bahia, Goiás and Minas Gerais States). In a preceding paper [80], the species was listed as troglobitic (referred to as *Eusarcus* sp. n. 3), but since it inhabits karstic areas that are not interconnected, these authors later interpreted the species as troglophile, or (alternatively) as representing an ensemble of ‘cryptic’ troglobites [79].

Harvestmen are important components of cave communities, with around 80 troglobitic species worldwide [2]. In South America, gonyleptoids considered strict troglobites comprised up to now 11 nominal species, from Brazil and Venezuela (compiled in Table 5). Most of them live in limestone caves, with only one Brazilian species found in a sandstone cave (Table 5). *Otilioleptes marcelae* gen. nov., sp. nov. joins the list as the twelfth troglobitic gonyleptoid, being the very first known from inside a lava tube. The degree of troglomorphism varies among the troglobitic gonyleptoids (Table 5). Of the species hitherto described only *Pachylospeleus strinatii*, *Iandumoema uai*, *I*. *smeagol*, *I*. *setimapocu* and *Giupponia chagasi* have eyes reduced or absent, and only the former has diffuse scutal grooves [15, 17, 18, 80, 81]. Some troglomorphic traits of *Otilioleptes marcelae* gen. nov., sp. nov., like depigmentation, little tegumentary sclerotization and faded mesotergal sulci, seem among the most accentuated in the superfamily; in other features, however, troglomorphy is not as outstanding (e.g., legs are among the less elongated, and tarsal count is also quite normal, except for the elongated basal tarsomere, see Table 5 and Fig 4C–4F).

**Table 5:** An account of the known troglobitic South American gonyleptoids (Opiliones, Laniatores) and their troglomorphic traits. Ratios of appendage length (femur length, in parentheses) over scutum length (or over total body length *) are based on measurements given in the literature, normally referred to the holotype; unavailable data indicated as “?”.

Species name	Author / reference	Family / Subfamily	Country: State or Province	Locality	Geology of cave	color	♂ appendage (femur) / scutum ratios	eyes cornea	retina
*Otilioleptes marcelae* gen. nov., sp. nov.	This paper	Otilioleptidae fam. nov.	Argentina: Mendoza	Cueva Doña Otilia, Malargüe	basalt	uniform whitish-yellowish, completely depigmented	Pp: 2.1 (0.6) Legs 3.3 (0.8) : 6.2 (1.5) : 4.3 (1.2) : 5.6 (1.6)	reduced to absent	spots of retinal pigment (deepened?)
*Pachylospeleus strinatii*	Šilhavý, 1974 [15]	Gonyleptidae / Pachylospeleinae	Brazil: São Paulo	Iporanga: Gruta das Areias de Cima, Gruta das Areias de Baixo, Ressurgência das Areias	limestone	light yellowish-red	[* on body length] Pp. 1.2 (0.4) Legs 3.2 (?) : 7.0 (?) : 4.1 (?) : 5.8 (?)	reduced	very narrow ring of black pigment only
*Iandumoema uai*	Pinto-da-Rocha, 1996 [17]	Gonyleptidae / Pachylinae	Brazil: Minas Gerais	Gruta Olhos d'Agua, Itacarambi	limestone	uniformly yellowish, depigmented; lateral margin, apophysis IV and trochanter-femur IV reddish-brown	Pp. 1.5 (0.5) Legs 4.4 (1.1) : 9.1 (2.1) : 5.0 (1.4) : 7.1 (2.0)	normal	pigments reduced
*Iandumoema setimapocu*	Hara & Pinto-da-Rocha, 2008 [80]	Gonyleptidae / Pachylinae	Brazil: Minas Gerais	Lapa do Zu cave, Coração de Jesus	limestone	uniformly light brown, depigmented; pedipalps, legs I–III and tibia–tarsus IV lighter	Pp. 1.6 (0.5) Legs 4.7 (1.0) : 9.6 (2.3) : 5.7 (1.6) : 8.0 (2.3)	reduced	depigmented
*Iandumoema smeagol*	Pinto-da-Rocha, Fonseca-Ferreira & Bichuette, 2015 [81]	Gonyleptidae / Pachylinae	Brazil: Minas Gerais	Monjolos, Toca do Geraldo cave; Lapa do Santo Antônio cave	limestone	pale yellowish carapace; tip of tarsus and dorsal tibia whitish	Pp. 1.7 (0.6) Legs 3.6 (1.1) : 7.1 (2.2) : 5.0 (1.5) : 6.9 (1.9)	absent	depigmented
*Giupponia chagasi*	Pérez & Kury, 2002 [18]	Gonyleptidae / Pachylinae	Brazil: Bahia	Serra do Ramalho, Carinhanha	limestone	depigmented	Pp. 2.6 (0.9) Legs 5.2 (1.3) : 10.2 (2.5) : 5.6 (1.6) : 7.4 (2.1)	absent	depigmented
*Eusarcus elinae*	Kury, 2008 [82]	Gonyleptidae / Pachylinae	Brazil: Bahia	Caverna Pedra Furada, Sistema Lapa Doce, Iraquara	limestone	lighter than in related epigean species (but not depigmented)	Pp. 1.1 (0.3) Legs 2.8 (0.7) : 6.4 (1.8) : 3.8 (1.1) : 6.6 (1.9)	normal	pigmented
*Discocyrtus pedrosoi*	Kury, 2008 [82]	Gonyleptidae / Pachylinae	Brazil: Bahia	Gruna do Brejo & Verruga, Andaraí, Distrito de Igatú	sandstone	lighter than in related epigean species (but not depigmented)	Pp. 2.1 (0.6) Legs 3.9 (0.9) : 8.3 (1.9) : 5.4 (1.3) : 6.2 (1.7)	normal	pigmented
*Spinopilar moria*	Kury & Pérez-González, 2008 [19]	Cryptogeobiidae	Brazil: Minas Gerais	Morena Cave, Cordisburgo	limestone	body and appendages uniform light mahogany brown	Pp. ? (?) Legs ? (0.9) : 7.9 (1.8) : ? (1.1) : ? (1.7)	normal	pigmented
*Trinella chapmani*	(Rambla, 1978) [83]	Agoristenidae	Venezuela: Falcón	Cueva de Trueno	limestone	much lighter than epigean species; pale straw-yellowish, chelicera, pedipalps, metatarsi and tarsi very light	[* on body length] Pp. 2.3 (0.6) Legs 7.9 (2.1) : 16.6 (4.2) : 10.1 (3.1) : 14.4 (4.5)	slightly reduced	pigmented
*Trinella bordoni*	(Muñoz-Cuevas, 1975) [16]	Agoristenidae	Venezuela: Zulia	Cueva Francisco Zea	limestone	yellowish	[* on body length] Pp. 1.8 (0.5) Legs 7.2 (1.9) : 15.3 (4.3) : 9.4 (2.7) : 13.3 (3.9)	absent	depigmented
*Trinella troglobia*	Pinto-da-Rocha, 1996 [84]	Agoristenidae	Venezuela: Zulia	Cueva de los Laureles; Cueva La Carlotica	limestone	yellowish, with scute margins and legs brownish (femur IV darker); pedipalps light	Pp. 1.9 (0.5) Legs 8.2 (2.1) : 16.7 (4.2) : 12.7 (2.8) : 13.4 (3.7)	absent in male, reduced in female	depigmented

**Note:** Records displayed by [80] as “*Eusarcus* sp. n. 1-2-3” and “Pachylinae sp. n. 1–2” are not included (*Eusarcus* sp. n. 1 was thereafter described as *E*. *elinae* [82]; *Euscarcus* sp. n. 2. as the troglophylic *E*. *cavernicola* Hara & Pinto-da-Rocha, 2010 [79]).

In sharp contrast with Brazil, reports of cave harvestmen are very scarce in Argentina, so it is worthwhile to add here some notes on them. These include the sparse “*Parabalta*” records from Las Brujas and Chorriaca (both limestone) [4], and unpublished material from “Aguada de la Mula” cave (gypsum), Neuquén Province (map in Fig 8), all three belonging to Gonyleptidae. They are the members of Gonyleptoidea geographically placed the nearest of *Otilioleptes* (Doña Otilia is 40 km from Las Brujas, 240 km from Chorriaca and Aguada de la Mula). Since all available samples consist of adult females, their generic assignment remains undefined. They all have in common that their troglomorphy is only insinuated by the slightly elongated appendages [4], but pigmentation and eye development display a decided epigean type. The modest troglomorphism of “*Parabalta*” suggests that these harvestmen might be ranked in either one of non-strict cave dwellers categories, troglophiles or trogloxenes [22, 78]. Epigean activity of harvestmen at Las Brujas was believed to be impossible due to the general aridity [4]; it was then hypothesized that these harvestmen were forced to stay inside the cave, despite their little specialization to cave life, and the expression ‘geographical troglophiles’ for this particular case was coined [4]. I was able to examine some amateur photos made by E. Chamorro during an occasional “harvestmen sighting” in Las Brujas, revealing two meaningful facts: males can be recognized by the well-developed sexual dimorphism (legs IV are armed, as usual in the family), and this species is probably not restricted to the cave but may have some kind of surface activity outside the cave. The inventory of Argentinean cave gonyleptids is completed by a record of *Discocyrtus testudineus* (Holmberg, 1876) from “Cueva de los Murciélagos”, northern Buenos Aires Province, a small cavity formed by erosion on the Paraná River cliffs [85]. Caves seem to represent an infrequent or accidental refuge for this epigean harvestman, which is common in the area and is widely spread in most of the ‘Mesopotamian’ opiliogeographical region [5, 86].

**Material examined.** ARGENTINA. *Mendoza Province*. Caverna de Las Brujas (Bardas Blancas, Malargüe), 1–5 Apr. 1985 (C. Benedetto—G.E.A.), 1 ♀ (MACN), *ca*. 35°47'S 69°49'W. *Neuquén Province*. Caverna de Chorriaca, 10 Feb. 1985 (G. Dejean), 1 ♀ (MACN), *ca*. 37°57'S 69°59'W; Caverna “Aguada de la Mula”, Cordón del Salado (gypsum cave, 30 m from entrance), 26 June 2000 (J.S. Romero & H. Cejas—Grupo Espeleológico del Neuquén), 1 ♀ (LEA 000.395), *ca*. 38° 3'S 70° 3'W.

## Discussion

The systematic assignment of *Otilioleptes* gen. nov. was difficult for several reasons, especially because of the astonishing simplicity of some external characters. This problem is not rare among troglobites. The systematic position of the troglomorph *Picunchenops spelaeus* is troublesome as well, and remained long unsolved: Maury [3] was unable to assign the genus to any subfamily, while Kury [63] listed it as ‘Triaenonychinae, Tribe uncertain’. As stressed, *Otilioleptes* has some unique features with little or no relationship to hypogean habits, the penis morphology in the first place. Genital traits usually are considered conservative and little influenced by cave-dwelling [87], what appears in agreement with the more or less ‘customary’ penial shapes shown by all other troglomorphic gonyleptoids [15, 17–19, 80]. Likewise, genital morphology of cave triaenonychids studied by [20] maintains a close resemblance to their epigean relatives. In two troglobitic gonyleptids in which troglomorphy is little accentuated (*Eusarcus elinae*, *Discocyrtus pedrosoi*), genitalia even allowed their easy assignment to extant epigean genera [82]. Although matching a generalized Laminata type well, the genitalic singularity of *Otilioleptes* depicts a puzzling gap with epigean harvestmen and is accordingly suspected to reflect a long-time isolation scenario, maybe this coupled with early divergence. Adaptation to cave seems unable to account for the almost lack of sexual dimorphism too. This is one outstanding difference with some relevant members of the superfamily, especially Gonyleptidae. With the apparent exception of *Giupponia chagasi*, in all hitherto known troglomorphic gonyleptids males and females are recognizable through the usual exomorphological dimorphism (Table 5). Finally, the coxa IV not completely fused to the stigmatic segment, and the latter extended beyond the coxa-trochanter joint (Fig 3B), are other peculiarities not linked to troglobitic adaptation. Despite their paucity, these features may contain valuable phylogenetic signals.

In an evolutionary context, two mutually exclusive scenarios might be invoked to understand these relationships and the morphological gap: either *Otilioleptes* diverged early from its relatives (and/or isolation in the cave operated with enough time, as to accumulate so many changes); or the species has undergone a rush evolutionary process. In the second option, relatives would be expected to be found among geographically neighboring taxa; nonetheless, to the moment no epigean relative of *Otilioleptes* has been recognized around or near the cave, and this might give little support to a rapid evolutionary process. In the alternative scenario (early divergence, long isolation), *Otilioleptes* would be relictual, and close relatives may have become extinct, or if still existing, they may be found anywhere. The basal position of the new genus in Laminata seems to sustain this scenario. It is generally accepted that high degree of troglomorphism is correlated with long isolation time [20] so that a long-term process seems at first sight better supported by all well-defined troglomorphic traits.

### Lava tubes and long-term evolution

However, an scenario of long-time evolution, as described above, appears to collide with the cave´s age. Albeit the exact chronology of Doña Otilia is unknown, the basaltic region itself is relatively young, estimated of Middle-Upper Pleistocene age (available datings near the cave range from 0.16 to 1 Mya [64, 65]). Such a short time seems a tight chronological constraint for large amounts of change. Moreover, volcanic caves seem a “poor choice” for long-term evolution, because they can only deteriorate, not grow as limestone caves do [6]. In fact, lava tubes have a rapid initial period of formation, then being degraded by erosion and siltation in a brief geologic time: there is no chance for enhancement of the main passage in a lava tube [22]. In any case, it is well known that troglobitic species can be older than the caves they inhabit [20, 88]. Lava tubes have plenty of fissures, cracks and crevices (the so-called “mesocavernous space” [89]), and these features may ultimately provide an effective connection with the intermediate-sized space known as MSS–*Milieu Souterrain Superficiel* [superficial subterranean environment] [90]. If new lava tubes are in a continuous process of formation, then the obligate cave dwellers might be able to migrate through the MSS into newly formed voids [6]. As stressed, two main extrusive events have been recorded in the Payunia region: a more recent Pliocene to Holocene event, younger than 5 Mya, and an older one, mostly Miocene in age (26–8 Mya; [64]), with throughout prevalence of monogenetic effusions. This might have offered a suitable background of smoothly evolving lava tubes, with enough time for long-term evolution, in which epigean relatives may have become extinct (hence the taxonomic gap).

### Influence of the epigean environmental changes

Together with the cave history, the evolution of the epigean landscape may provide additional, coarse clues on the origin of *Otilioleptes*. Paleoenvironmental studies demonstrated that conditions in the Patagonian and Monte ecoregions were very different in the past. Inspired on Ringuelet (1978) [10], Maury (1986) [4] explained the presence of “*Parabalta*” in Las Brujas and Chorriaca caves by the former extension of the ‘subtropical’ biota, which retreated northwards as aridity increased after the rise of the Andes; in this view, these harvestmen would represent relics of subtropical origin. A comparable relictual condition, involving withdrawal of ancestral forests, has been suggested for other cave gonyleptoids in Brazil [19, 82]. This scenario is consistent with the ‘Climatic Relict Hypothesis’ proposed by Thomas Barr in the 1960s, in which the parental epigean populations become extinct because of climatic change, leaving relictual survivors in the cave [88].

At this point, it would be of interest to determine how long ago, if ever, ‘subtropical’ (or at least humid) conditions might have existed around Doña Otilia. Based on the absence of glacial erosion in the Payunian volcanos, the present aridity was extrapolated at least up to the Late Pleistocene [64]; but very likely similar xeric vegetation extended back over the Pliocene, up to the Late Miocene [91, 92]. During the Miocene, the strong Andean uplift (especially during the ‘Quechua diastrophic phase’, 14–10 Mya), combined with the fall in global temperature, determined the establishment of arid and markedly seasonal environments east of the Cordillera, as they look today [93, 94]. Xeric epigean conditions then seem the presumed frame for the younger Payunian volcanic events (from 5.1 Mya [64]). For North American cave sclerobunines (Laniatores: Travuniidae) it was estimated that 5 Myr might be correlated with only moderate troglomorphy; highly troglomorphic taxa might need at least 10 Myr divergence time to evolve [20]. Regardless of those appraisals (made on distant taxa, and in a different geographical context, indeed), an epigean ‘subtropical’ ancestor wandering on the arid surface at those times sounds hardly credible. Further backwards, a period of volcanic inactivity has been reported in the Payunia from 8 to 5 Mya [64] (Late Miocene); for this period, small thorny trees and bushes covering most of the Patagonia west of the Andes were inferred [95, 96]. One has to go back to Early-Middle Miocene–i.e., before climate started to differentiate both sides of the rising Andes–to find the final expressions of mixed subtropical conditions in parts of Patagonia [91, 95, 96]. They represent the remnants of preceding forests that extensively covered the area, in the form of subtropical (megathermal) rainforests in the Paleocene–Early Eocene, and with the emergence of meso- and microthermal communities (including *Nothofagus*) in Middle Eocene–Oligocene [91, 92]. Environmental changes in the Miocene were not just limited to climate: successive marine transgressions, collectively referred to as the ‘Paranean Sea’, submerged at ~15–13 Mya large portions of Patagonia and central Argentina [91], though likely not the Payunia [97]. The Paranean Sea was hypothesized to be part of an intracontinental seaway that separated the Andean-Patagonian from the extra-Andean realms [91, 98]. Early-Middle Miocene (and final Upper Oligocene) are the ages for the older Payunian volcanic stage, spanning over an extended time lapse between 26 and 8 Mya [64]. These rough correspondences might vaguely place the divergence from a putative subtropical ancestor sometime in the referred periods, likely around Middle Miocene. Such a chronology appears consistent with the basal condition of *Otilioleptes*, as suggested by the cladograms. It is not known, however, if lava caves themselves or the associated MSS could have persisted during the prolonged 3 Myr volcanic inactivity in Late Miocene [64] as to enable the survival of the troglobites into the Pliocene. It cannot be discarded that the hypogean condition of *Otilioleptes* originated elsewhere (for example, in neighboring karst cavities, or in the MSS), then migrating into the lava tubes more recently.

Suggestively, the same events deemed to have isolated *Otilioleptes* (rise of the Andes, increased aridity in western Argentina, eventually the Paranean Sea) might have well been responsible for the definitive separation of the Chilean gonyleptid fauna from its subtropical ‘source’. During the referred periods, especially from Middle Miocene onwards, the Payunia seems to have maintained broader and more continuous contacts with the Chilean biota than with the subtropical one (cf. [99]). Hence, a most recent common ancestry of *Otilioleptes* with a Chilean gonyleptoid, rather than with a ‘pure’ subtropical lineage, emerges as a robust alternative hypothesis. The cladistic vicinity with the Chilean genus *Osornogyndes* might give support to this scenario, although evidence for *Osornogyndes* and *Otilioleptes* sharing a presumed common ancestor is, to the moment, poor. Similarly, the Chilean origin for *Picunchenops spelaeus* was proposed [3], in this case suggesting that subantarctic vegetation may have shifted northwards, presumably driven by Pleistocene glacier expansions, to leave isolates in the cave in the retreat stage (the latter also combined with the increasing aridity after the Miocene). As seen, a least for *Otilioleptes*, divergence time is probably much older than the Pleistocene, so that Maury´s [3] approach does not seem fully applicable for the new genus.

## Concluding remarks

In sum, with the scattered evidence put together (degree of troglomorphism; some unique features not attributable to cave adaptations; the basal and isolated cladistic placement within Laminata; geologic and paleoenvironmental background), *Otilioleptes* might be hypothesized to be a relictual member of an early gonyleptoid lineage, which survived in evolving lava tubes or associated cavities since long ago. Despite having only a weak hypothesis on the closest relatives of *Otilioleptes*, the dilemma, subtropical vs Chilean origin of the new genus appears better supported for the second option. Testing these scenarios would represent a challenge for future research, which should start by inquiring in more depth the riddle of the phylogenetic relationships of *Otilioleptes* and *Osornogyndes*. Molecular analyses are foreseen to test the phylogenetic hypothesis obtained here. It is expected that molecular data might help overcome the limitations of a purely morphological approach, especially those derived from the marked troglomorphism and the high proportion of presumable plesiomorph states.

There is also an anecdotal side around Doña Otilia: the cave was named after the grandmother of Mr Martín Zagal, who first discovered this lava tube [72]. But also the roots of the name *Otilia* (German: *Ottilien*, female diminutive of the medieval given name Otto), meaning "rich, wealthy or prosperous", may inspire a portrayal of the biological richness and evolutionary value of the cave. Beyond those allegories, the uniqueness of this lava tube, as well as the fragility and vulnerability of its hypogean ecosystem emerge as clear-cut conclusions and should stimulate further biospeleological research, along with the implementation of effective measures for its adequate preservation. At present knowledge, conservation priority of Doña Otilia cave seems undoubtedly the highest.

## Supporting information

S1 Table Matrix of 85 effective characters x 45 terminals, used in the cladistic and Bayesian analyses of Gonyleptoidea to assess affinities of *Otilioleptes* gen. nov. (DOC)

S1 Text Characters, character states and coding, as applied in the matrix of S1 Table. (PDF)

S1 Fig Summary of cladistic relationships of *Otilioleptes* gen. nov. and the main clades within Laminata (L), in the different analytical treatments performed in this paper. (PDF)

S2 Fig Cladistic relationships of Gonyleptoidea and *Otiloleptes marcelae* gen. nov., sp. nov.: Character optimization on the selected tree (IW, k = 6). (PDF)

S3 Fig Bayesian analysis of Gonyleptoidea and *Otiloleptes marcelae* gen. nov., sp. nov. (PDF)

## References

[1] White WB. 2005 Volcanic caves In: Culver DC, White WB, eds. *Encyclopedia of Caves*. Elsevier, 599–602.

[2] Culver DC, Pipan T. 2009 *The biology of caves and other subterranean habitats* Oxford: Oxford University Press.

[3] Maury EA. 1988 Triaenonychidae sudamericanos. V. Un nuevo género de Opiliones cavernícolas de la Patagonia (Opiliones, Laniatores). *Mémoires de Biospéologie* 15: 117–131.

[4] Maury EA. 1986 Hallazgo aracnológico en cavernas del oeste argentino. *Salamanca* 2(2): 20–24.

[5] Acosta LE. 2002 Patrones zoogeográficos de los opiliones argentinos (Arachnida: Opiliones). *Revista Ibérica de Aracnología* 6: 69–84.

[6] Briggs TS. 1974 Troglobitic harvestmen recently discovered in North American lava tubes (Travuniidae, Erebomastridae, Triaenonychidae: Opiliones). *The Journal of Arachnology* 1: 205–214.

[7] Ringuelet RA. 1957 Biogeografía de los arácnidos argentinos del Orden Opiliones. *Contribuciones Científicas*, *Serie Zoología*, *Facultad de Ciencias Exactas y Naturales*, Universidad de Buenos Aires 1(1): 1–33.

[8] Ringuelet RA. 1959 Los arácnidos argentinos del Orden Opiliones. Revista del Museo Argentino de Ciencias Naturales, Ciencias Zooló*gicas* 5(2): 127–439, Pl. I–XX.

[9] Ringuelet RA. 1962 Un nuevo opilión de fauna de altura y observaciones sobre vinculaciones evolutivas en algunos Pachylinae (Arachnida). *Revista de la Sociedad Entomológica Argentina* 23(1–4): 1–6.

[10] Ringuelet RA. 1978 Dinamismo histórico de la fauna brasílica en la Argentina. *Ameghiniana* 15(1–2): 255–262.

[11] Maury EA, Roig Alsina AH. 1982 Sobre la presencia de Opiliones en las provincias argentinas de Mendoza y San Juan (Arachnida, Opiliones). *Neotrópica* 28(79): 39–40.

[12] Maury EA, Roig Alsina AH. 1985 Triaenonychidae sudamericanos. I. El género Ceratomontia Roewer 1915 (Opiliones: Laniatores). *Historia Natural* 5(11): 77–92.

[13] Benedetto C. 1999 Volcanic caves in Argentina. *Proceedings of the IXth International Symposium on Vulcanospeleology*, pp. 219–222.

[14] Benedetto C, Peralta M. 2007 Observaciones sobre la ecología de la Cueva Doña Otilia (Malargüe, Mendoza, Argentina). *I Congreso de la Federación Espeleológica de Puerto Rico (FEPUR)—V Congreso de la Federación Espeleológica de América Latina y del Caribe (FEALC)*, Aguadilla (Puerto Rico); *Focus* 6(1–2): 162–163.

[15] Šilhavý V. 1974 A new subfamily of Gonyleptidae from Brazilian caves, Pachylospeleinae subf. n. (Opiliones, Gonyleptomorphi). *Revue Suisse de Zoologie* 81(4): 893–898.

[16] Muñoz-Cuevas A. 1975 Phalangozea bordoni, nuevo género y especie de opiliones cavernícolas de Venezuela, de la familia Phalangodidae (Arachnida: Opilionida). *Boletín de la Sociedad Venezolana de Espeleología* 6(12): 87–94.

[17] Pinto-da-Rocha R. 1996 Iandumoema uai, a new genus and species of troglobitic harvestman from Brazil (Arachnida, Opiliones, Gonyleptidae). *Revista Brasileira de Zoologia* 13(4): 843–848.

[18] Pérez [González] A, Kury AB. 2002 A new remarkable troglomorphic gonyleptid from Brazil (Arachnida, Opiliones, Laniatores). *Revista Ibérica de Aracnología* 5: 43–50.

[19] Kury AB, Pérez-González A. 2008 The first cave-dwelling Spinopilar Mello-Leitão 1940 (Opiliones Gonyleptidae Tricommatinae), described from a Brazilian cave. *Tropical Zoology* 21: 259–267.

[20] Derkarabetian S, Steinmann DB, Hedin M. 2010 Repeated and time-correlated morphological convergence in cave-dwelling harvestmen (Opiliones, Laniatores) from montane western North America. *PLoS ONE* 5(5): e10388 10.1371/journal.pone.0010388 20479884 PMC2866537

[21] Pérez-González A, Ceccarelli FS, Monte BGO, Proud DN, DaSilva MB, Bichuette ME. 2017 Light from dark: A relictual troglobite reveals a broader ancestral distribution for kimulid harvestmen (Opiliones: Laniatores: Kimulidae) in South America. PLoS ONE 12(11): e0187919 10.1371/journal.pone.0187919 29190302 PMC5708626

[22] Howarth FG. 1973 The cavernicolous fauna of Hawaiian lava tubes, 1. Introduction. *Pacific Insects* 15(1): 139–151.

[23] Kury AB. 2014 Why does the Tricommatinae position bounce so much within Laniatores? A cladistic analysis, with description of a new family of Gonyleptoidea (Opiliones, Laniatores). *Zoological Journal of the Linnean Society* 172: 1–48.

[24] Maury EA. 1993 Gonyleptidae (Opiliones) del bosque subantártico chileno-argentino. III. Descripción de Osornogyndes, nuevo género. *Boletín de la Sociedad de Biología de Concepción* 64: 99–104.

[25] Kury AB, Villarreal M O. 2015 The prickly blade mapped: establishing homologies and a chaetotaxy for macrosetae of penis ventral plate in Gonyleptoidea (Arachnida, Opiliones, Laniatores). *Zoological Journal of the Linnean Society* 174: 1–46.

[26] Sharma PP, Giribet G. 2011 The evolutionary and biogeographic history of the armoured harvestmen–Laniatores phylogeny based on ten molecular markers, with the description of two new families of Opiliones (Arachnida). *Invertebrate Systematics* 25: 106–142.

[27] Pinto-da-Rocha R., Bragagnolo C., Marques FPL, Antunes Junior M. 2014 Phylogeny of harvestmen family Gonyleptidae inferred from a multilocus approach (Arachnida: Opiliones). *Cladistics* 30: 519–539. 10.1111/cla.12065 34772271

[28] Bragagnolo C, Hara MR, Pinto-da-Rocha R. 2015 A new family of Gonyleptoidea from South America (Opiliones, Laniatores). *Zoological Journal of the Linnean Society* 173: 296–319.

[29] Mendes AC & Kury AB. 2012 Notes on the systematics of the Triaenonychinae from Madagascar with description of new species of Acumontia Loman (Opiliones: Laniatores). *Zootaxa* 3593: 40–58.

[30] Pérez González A. 2006. Revisão sistemática e análise filogenética de Stygnommatidae (Arachnida, Opiliones). Ph. D. Thesis, Universidade Federal do Rio de Janeiro.

[31] Cruz-López JA, Francke OF. 2013 Two new species of the genus Paramitraceras Pickard-Cambridge, 1905 (Opiliones: Laniatores: Stygnopsidae) from Chiapas, Mexico. *Zootaxa* 3641 (4): 481–490. 26287101 10.11646/zootaxa.3641.4.13

[32] Pinto-da-Rocha R & Hara MR. 2009 New familial assignments for three species of Neotropical harvestmen based on cladistic analysis (Arachnida: Opiliones: Laniatores). *Zootaxa* 2241: 33–46.

[33] Pinto-da-Rocha R. 1997 Systematic review of the Neotropical Family Stygnidae (Opiliones, Laniatores, Gonyleptoidea). *Arquivos de Zoologia* 33(4): 163–342.

[34] Kury AB, Maury EA. 1998 A new genus and five new species of Metasarcinae from Peru (Arachnida, Opiliones, Gonyleptidae). *Zoological Journal of the Linnean Society* 123: 143–162.

[35] Kury AB, Villarreal Manzanilla O, Sampaio C. 2007 Redescription of the type species of Cynorta (Arachnida, Opiliones, Cosmetidae). *The Journal of Arachnology* 35: 325–333.

[36] Kury AB. 1997 The genera *Saramacia* and *Syncranaus* Roewer, with notes on the status of the Manaosbiidae (Opiliones, Laniatores, Gonyleptoidea). *Boletim do Museu Nacional, Nova Série, Zoología* (374): 1–22.

[37] Kury AB. 2012 A new genus of Cranaidae from Ecuador (Opiliones: Laniatores). *Zootaxa* 3314: 31–44.

[38] Hara MR, Pinto-da-Rocha R, Villarreal M. O. 2014 Revision of the cranaid genera Phalangodus, Iquitosa and Aguaytiella (Opiliones: Laniatores: Gonyleptoidea). *Zootaxa* 3814 (4): 567–580. 10.11646/zootaxa.3814.4.8 24943449

[39] Villarreal M. O & Kury AB. 2012 Licornus Roewer, 1932: newly transferred to Ampycinae and first record of the family Gonyleptidae (Opiliones: Laniatores) from Venezuela. *Zootaxa* 3544: 71–78.

[40] García AF. 2014 Primeros registros de Ampycinae Kury, 2003 (Opiliones, Gonyleptidae) en Colombia. *Revista Ibérica de Aracnología* 25: 93–95.

[41] Acosta LE. 1989 Pachyloides hades, nueva especie de opilión de la Argentina (Opiliones, Gonyleptidae, Pachylinae). *The Journal of Arachnology* 17(1): 137–142.

[42] DaSilva MB, Gnaspini P. 2009 A systematic revision of Goniosomatinae (Arachnida: Opiliones: Gonyleptidae), with a cladistic analysis and biogeographical notes. *Invertebrate Systematics* 23: 530–624.

[43] Weber M. 1988. Die Phalangodidae—eine polyphyletische Familie der Gonyleptoidea? (Arachnida: Opiliones: Laniatores). Diplomarbeit (BSc thesis), Fakultät für Biologie, Universität Tübingen. Available at: http://www.mwspider.de/Publication/diplom.htm

[44] Kury AB. 2003 A new species of Pherania Strand, 1942 from southern Brazil (Arachnida: Opiliones: Gonyleptidae). *Zootaxa* 363: 1–8.

[45] Kury AB. 2002 A new genus of Tricommatinae from Eastern Brazil (Opiliones Laniatores Gonyleptidae). *Tropical Zoology* 15: 209–218.

[46] Goloboff PA, Farris JS, Nixon KC. 2008 TNT, a free program for phylogenetic analysis. *Cladistics* 24: 774–786.

[47] Goloboff PA. 1993 Estimating character weights during tree search. *Cladistics* 9: 83–91. 10.1111/j.1096-0031.1993.tb00209.x 34929936

[48] Nixon KC. 1999–2002. *Winclada*, ver. 1.00.08 Published by the author, Ithaca, NY, USA.

[49] Bremer K. 1994 Branch support and tree stability. *Cladistics* 10: 295–304.

[50] Lewis PO. 2001 A likelihood approach to estimating phylogeny from discrete morphological character data. *Systematic Biology* 50(6): 913–925. 10.1080/106351501753462876 12116640

[51] Wright AM. 2019 A systematist’s guide to estimating Bayesian phylogenies from morphological data. *Insect Systematics and Diversity* 3(3): 2; 1–14. 10.1093/isd/ixz006 31355348 PMC6643758

[52] Wright AM, Hillis DM. 2014 Bayesian analysis using a simple likelihood model outperforms parsimony for estimation of phylogeny from discrete morphological data. *PLoS ONE* 9(10): e109210 10.1371/journal.pone.0109210 25279853 PMC4184849

[53] O’Reilly JE, Puttick MN, Pisani D, Donoghue PCJ. 2018 Probabilistic methods surpass parsimony when assessing clade support in phylogenetic analyses of discrete morphological data. *Palaeontology* 61 (1): 105–118. 10.1111/pala.12330 29398726 PMC5784394

[54] Sansom RS, Choate PG, Keating JN, Randle E. 2018 Parsimony, not Bayesian analysis, recovers more stratigraphically congruent phylogenetic trees. *Biology Letters* 14: 20180263 10.1098/rsbl.2018.0263 29925561 PMC6030593

[55] Goloboff PA, Torres A, Arias JS. 2018 Weighted parsimony outperforms other methods of phylogenetic inference under models appropriate for morphology. *Cladistics* 34(4): 407–437. 10.1111/cla.12205 34649370

[56] Goloboff PA, Pittman M, Pol D, Xu X. 2019 Morphological data sets fit a common mechanism much more poorly than DNA sequences and call into question the Mkv model. *Systematic Biology* 68(3): 494–504. 10.1093/sysbio/syy077 30445627

[57] Ronquist F, Huelsenbeck JP. 2003 MrBayes 3: Bayesian phylogenetic inference under mixed models. *Bioinformatics* 19: 1572–1574. 10.1093/bioinformatics/btg180 12912839

[58] Acosta LE, Pérez González A, Tourinho AL. 2007 Methods for taxonomic study In: Pinto-da-Rocha R, Machado G, Giribet G, eds. *Harvestmen: The Biology of Opiliones*. Cambridge, Massachusetts: Harvard University Press, 494–505.

[59] Olson DM, Dinerstein E, Wikramanayake ED, Burgess ND, Powell GVN, Underwood EC et al 2001 Terrestrial ecoregions of the world: a new map of life on Earth. *BioScience* 51: 933–938. Shapefile “Terrestrial Ecoregions of the World—Version 2.0”, published 2004 by World Wildlife Fund; available at http://worldwildlife.org/publications/terrestrial-ecoregions-of-the-world

[60] Giribet G, Vogt L, Pérez González A, Sharma P, Kury AB. 2010 A multilocus approach to harvestman (Arachnida: Opiliones) phylogeny with emphasis on biogeography and the systematics of Laniatores. *Cladistics* 26: 408–437. 10.1111/j.1096-0031.2009.00296.x 34875803

[61] Kury AB. 2009 Infraorder Grassatores. In: Kury AB(Ed.), Project Opilionomicon. Museu Nacional, Rio de Janeiro. Online at: http://www.museunacional.ufrj.br/mndi/ Aracnologia/Opilionomicon/Infraorder Grassatores.htm

[62] Kury AB. 2015 Opiliones are no longer the same—on suprafamilial groups in harvestmen (Arthropoda: Arachnida). *Zootaxa* 3925 (3): 301–340. 10.11646/zootaxa.3925.3.1 25781747

[63] Kury AB. 2003 Annotated catalogue of the Laniatores of the New World (Arachnida, Opiliones). *Revista Ibérica de Aracnología, Volumen especial monográfico* 1: 5–337.

[64] Llambías EJ, Bertotto GW, Risso C, Hernando I. 2010 El volcanismo cuaternario en el retroarco de Payenia: una revisión. *Revista de la Asociación Geológica Argentina* 67(2): 278–300.

[65] Gudnason J, Holm PM, Søager N, Llambías EJ. 2012 Geochronology of the late Pliocene to recent volcanic activity in the Payenia back-arc volcanic province, Mendoza Argentina. *Journal of South American Earth Sciences* 37: 191–201.

[66] Søager N, Holm PM, Llambías EJ. 2013 Payenia volcanic province, southern Mendoza, Argentina: OIB mantle upwelling in a backarc environment. *Chemical Geology* 349–350: 36–53.

[67] Hernando IR, Llambías EJ, González PD, Sato K. 2012 Volcanic stratigraphy and evidence of magma mixing in the Quaternary Payún Matrú volcano, Andean backarc in western Argentina. *Andean Geology* 39: 158–179.

[68] Candia R, Puig S, Dalmasso E, Videla F, Martínez Carretero E. 1993 Diseño del plan de manejo para la Reserva Provincial La Payunia (Malargüe, Mendoza). *Multequina* 2: 5–87.

[69] Norte F. 2000 Mapa climático de Mendoza. *Catálogo de recursos humanos e información relacionada con la temática ambiental en la región andina argentina*. Caracterización general y estudios temáticos por Provincia. Laboratorio de Desertificación y Ordenamiento Territorial. IADIZA. http://www.cricyt.edu.ar/ladyot/catalogo/cdandes/cap04.htm (accessed 20 May 2012).

[70] Roig FA, Martínez Carretero E, Méndez E. 2000 Vegetación de la Provincia de Mendoza. *Catálogo de recursos humanos e información relacionada con la temática ambiental en la región andina argentina*. *Caracterización general y estudios temáticos por Provincia*. Laboratorio de Desertificación y Ordenamiento Territorial. IADIZA. http://www.cricyt.edu.ar/ladyot/catalogo/cdandes/cap04.htm (accessed 20 May 2012).

[71] Paruelo JM, Golluscio RA, Jobbágy EG, Canevari M, Aguiar MR. 2005 Situación ambiental en la estepa patagónica In: Brown A, Martínez Ortiz U, Acerbi M, Corcuera J, eds. *La situación ambiental argentina 2005*. Buenos Aires: Fundación Vida Silvestre Argentina, 303–313.

[72] Brojan M. 2000 Biología en Cueva Doña Otilia (Malargüe, Mendoza, Argentina). *Spelaion* 7: 55–58.

[73] Mikkan R. 2014 Payunia, Campos Volcánicos Llancanelo y Payún Matrú: Patrimonio Mundial. *Tiempo y Espacio* 33: 31–47.

[74] Gnaspini P. 1996 Population ecology of Goniosoma spelaeum, a cavernicolous harvestman from south-eastern Brazil (Arachnida: Opiliones: Gonyleptidae). *Journal* of Zoology, London 239: 417–435.

[75] Pinto-da-Rocha R. 1996 Description of the male of Daguerreia inermis Soares & Soares, with biological notes on population size in the Gruta da Lancinha, Paraná, Brazil (Arachnida, Opiliones, Gonyleptidae). *Revista Brasileira de Zoologia* 13(4): 833–842.

[76] Machado G, Raimundo RLG, Oliveira PS. 2000 Daily activity schedule, gregariousness, and defensive behaviour in the Neotropical harvestman Goniosoma longipes (Opiliones: Gonyleptidae). *Journal of Natural History* 34: 587–596.

[77] Santos FH, Gnaspini P. 2002 Notes on the foraging behavior of the Brazilian cave harvestman Goniosoma spelaeum (Opiliones, Gonyleptidae). *The Journal of Arachnology* 30: 177–180.

[78] Gnaspini P, Hoenen S. 1999 Considerations about the troglophilic habit: the cave cricket model. *Mémoirs de Biospéologie* 26: 151–158.

[79] Hara MR, Pinto-da-Rocha R. 2010 Systematic review and cladistic analysis of the genus Eusarcus Perty 1833 (Arachnida, Opiliones, Gonyleptidae). *Zootaxa* 2698: 1–136.

[80] Hara MR, Pinto-Da-Rocha R. 2008 A new species of Brazilian troglobitic harvestman of the genus Iandumoema (Opiliones: Gonyleptidae). *Zootaxa* 1744: 50–58.

[81] Pinto-da-Rocha R, Fonseca-Ferreira R, Bichuette ME. 2015 A new highly specialized cave harvestman from Brazil and the first blind species of the genus: Iandumoema smeagol sp. n. (Arachnida, Opiliones, Gonyleptidae). *ZooKeys* 537: 79–95. 10.3897/zookeys.537.6073 PMC4714048 26798238

[82] Kury AB. 2008 Two new troglomorph Pachylinae (Opiliones, Laniatores, Gonyleptidae) from caves in Bahia, Brazil. *Studies on Neotropical Fauna and Environment* 43(3): 247–253.

[83] Rambla M. 1978 Opiliones cavernícolas de Venezuela (Arachnida, Opiliones Laniatores). *Speleon* 24: 5–22.

[84] Pinto-da-Rocha R. 1996 Notes on Vima insignis Hirst, 1912, revalidation of Trinella Goodnight & Goodnight, 1947 with description of three new species (Arachnida, Opiliones, Agoristenidae). *Revista Brasileira de Entomologia* 40(2): 315–323.

[85] Lipps E, Austin J, Pérez González A. 2006 Observaciones biológicas en la “Cueva de los Murciélagos”. Vuelta de Obligado, provincia de Buenos Aires, República Argentina In: Mérida E, Athor J, eds. *Talares bonaerenses y su conservación*. Buenos Aires: Fundación de Historia Natural “Félix de Azara”, 178–179.

[86] Acosta LE. 2014 Bioclimatic profile and potential distribution of the Mesopotamian harvestman Discocyrtus testudineus (Holmberg, 1876) (Opiliones, Gonyleptidae). *Zootaxa* 3821(3): 301–320. 10.11646/zootaxa.3821.3.1 24989746

[87] Hedin M, Thomas SM. 2010 Molecular systematics of eastern North American Phalangodidae (Arachnida: Opiliones: Laniatores), demonstrating convergent morphological evolution in caves. *Molecular Phylogenetics and Evolution* 54: 107–121. 10.1016/j.ympev.2009.08.020 19699807

[88] Wessel A, Erbe P, Hoch H. 2007 Pattern and process: Evolution of troglomorphy in the cave-planthopper of Australia and Hawaii–preliminary observations (Insecta: Hemiptera: Fulgoromorpha: Cixiidae). *Acta Carsologica* 36: 199–206.

[89] Stone FD, Howarth FG, Hoch H, Asche M. 2005 Root communities in lava tubes In: Culver DC, White WB, eds. *Encyclopedia of Caves*. Elsevier, 477–484.

[90] Juberthie C, Delay B, Bouillon M. 1980 Extension du milieu souterrain en zone non-calcaire: description d´un noveau milieu et de son peuplement par les coleoptères troglobies. *Mémoirs de Biospéologie* 7: 19–52.

[91] Ortiz-Jaureguizar E, Cladera GA. 2006 Paleoenvironmental evolution of southern South America during the Cenozoic. *Journal of Arid Environments* 66: 498–532.

[92] Barreda V, Palazzesi L. 2007 Patagonian vegetation turnovers during the Paleogene-Early Neogene: origin of arid-adapted floras. *The Botanical Review* 73: 31–50.

[93] Iglesias A, Artabe AE, Morel EM. 2011 The evolution of Patagonian climate and vegetation from the Mesozoic to the present. *Biological Journal of the Linnean Society* 103: 409–422.

[94] Palazzesi L, Barreda V. 2012 Fossil pollen records reveal a late rise of open-habitat ecosystems in Patagonia. *Nature Communications* 3: 1294 10.1038/ncomms2299 23250424

[95] Barreda V, Anzótegui LM, Prieto AR, Aceñolaza P, Bianchi MM, Borromei AM et al 2007 Diversificación y cambios de las angiospermas durante el Neógeno en Argentina. *Asociación Paleontológica Argentina, Publicación Especial* (*Ameghiniana 50º aniversario*) 11: 173–191.

[96] Palazzesi L, Barreda V. 2007 Major vegetation trends in the Tertiary of Patagonia (Argentina): A qualitative paleoclimatic approach based on palynological evidence. *Flora* 202: 328–337.

[97] Malumián N, Náñez C. 2011 The Late Cretaceous–Cenozoic transgressions in Patagonia and the Fuegian Andes: foraminifera, palaeoecology, and palaeogeography. *Biological Journal of the Linnean Society* 103: 269–288.

[98] Hulka C, Gräfe K-U, Sames B, Uba CE, Heubeck C. 2006 Depositional setting of the Middle to Late Miocene Yecua Formation of the Chaco Foreland Basin, southern Bolivia. *Journal of South American Earth Sciences* 21: 135–150.

[99] Hinojosa LF, Villagrán C. 1997 Historia de los bosques del sur de Sudamérica, I: Antecedentes paleobotánicos, geológicos y climáticos del Terciario del cono sur de América. *Revista Chilena de Historia Natural* 70: 225–239.

