# Supplementary material for: A relictual troglomorphic harvestman discovered in a volcanic cave of western Argentina: Otilioleptes marcelae, new genus, new species, and Otilioleptidae, new family (Arachnida, Opiliones, Gonyleptoidea)
Source: PLoS One. 2019 Oct 23;14(10):e0223828. doi: 10.1371/journal.pone.0223828 (PMC6808334; doi:10.1371/journal.pone.0223828)
Supplement: S1 Table — (DOC) [file pone.0223828.s001.doc]

**S1 Table. Matrix of 85 effective characters x 45 terminals, used in the cladistic analysis of Gonyleptoidea to assess affinities of *Otilioleptes* gen. nov.** Character coding: see S1 Text. Deactivated(·): 23, 48, 62, 63, 64.

---------1---------2---------3---------4---------5---------6---------7---------8---------9

123456789012345678901234567890123456789012345678901234567890123456789012345678901234567890

1. TRIAE_Acumontia_succinea 00000000002-0000000002·000020000000000000300-00·0-000000-0000···-------?--0------110?01101

2. STGNOM_Stygnomma_fuhrmanni 000-1000002-0010010000·0000?0000000000101001-00·0-000000-0000···-------?--0------110300000

3. POD_gen._sp. [68]0000001212-2010210012·000000000100000101001-00·0-00000000000···0?102-??11010010-111101101

4. ASS_Maracandellus_sp. 6101010000000000100201·0100?0000100000101001-0-·0-00020000000···001010???0100201-210100101

5. ASS_Ayenea_trimaculata [56]101012100010000100001·010000000000000101001--0·0-00023000000···---110230012??00-111100200

6. EPE_Metepedanulus_cf._flaveolus [68]101000000000011100012·000010000000000101001--0·0-00020000000···1000014000110211-100300100

7. PYR_Pyramidops_pygmaeus 5100000000000100000012·000000000102000101001-00·0-00020000000···1500100000110011-111310020

8. STOPS_Paramitraceras_granulatum 5000000000020020000012·00002000-102100101001-00·0-00020000000···100025?000100202-110110101

9. AGO_Globibunus_rubrofemoratus 1100001000000100000312·000000100100101101102-00·0002010000000···1000--3000111121-111121310

10. STY_Ricstygnus_quineti 511-101100020?20000000·000010000021111111102-00·0001010000001···1000141301112001-1??3?????

11. STY_Stygnus_mediocris 101-101100040120000000·000010110020111111102010·0001010000000···1000141401112001-111300111

12. CRYP_Zalanodius_convexus 50-0000000000100-00000·0100000-0101?-1101102-00·0000110000000···10002011001111113-?0110232

13. CRYP_Spinopilar_moria 6101000000000000100000·01000000010000?101102000·0000110000000···100020--001111113101200112

14. CRYP_Cryptogeobius_crassipes 2100010000000000100010·010000011121111101102000·0003110000000···10002011011111113110200132

15. GER_Gerdesius_mapinguari 6100010000030100100000·000000010122101111102-00·0003010000001···100022?001111111-101100222

16. NOM_Quindina_albomarginis 3101001100122100100010·000000000000001111203011·0000014100000···0601250201112011-111101301

17. NOM_Nomoclastes_quasimodo 50--1020002-0030000000·000000000002001111203011·0000014100000···0601250201112001-110110100

18. NOM_Zamora_sp. 5010001000020020000002·010000100001111111203000·0001014100001···020123141111?010-110110000

19. MET_Incasarcus_dianae 2101011100100100000012·000020100100101101203000·1000010101101···0400251411120012-101100310

20. MET_Metasarcus_sp 2101011110000100100012·010030100120101101203000·120101010110?···0400251411110011-111000311

21. MET_gen.sp. 3001002100100000100012·000030000100001101203000·100001010110?···0400252311110010-111000310

22. COS_Cynorta_conspersa 31010021002-0001101101·101040000000001111203000·0001010002101···0400251411110012-101000310

23. COS_Gryne_orensis 31010021002-0101101101·101040000000101111203000·0001010002101···0400231411110012-101010310

24. COS_Gnidia_holmbergii 21010021002-0001101101·1-1040000020101111203000·0001010002101···040025141111?012-101010310

25. MAN_Syncranaus_cribrum 31010011002-0000100010·000031010020001111203000·0000010100001···0400252311110011-1?1100230

26. MAN_Saramacia_lucasae 31010011002-0000100010·000031010020001111203000·0000010100001···0400252311110011-111?10210

27. CRA_Chiriboga_albituber 60110011002-0020000002·000020010020101111213100·0200011110011···0411251311112012-101110011

28. CRA_Zannicranaus_monoclonius 40110131002-0020200002·000120010020101111213100·0203011110011···0410251311120012-201121110

29. CRA_Phalangodus_sp 2101003100000000200003·000020010000101111213100·0203010110001···040025?311101012-101121110

30. AMP_Licornus_tama 4101011110031100100000·00003001111211111120300?·020101210000?···040025?301112010-10110022?

31. AMP_Hutamaia_caramaschii 4101011020031?00100000·000030011112111111203000·0200012100000···0400252301111012-1?1100211

32. AMP_Ampycus_telifer 21000111112-1100100000·000030011122111111203000·0210012100001···0400252301110010-101000220

33. GON_Discocyrtus_testudineus 4201011100012100100010·000030311122111111203000·0200110000001···04002514111100110101100222

34. GON_Gonyleptes_horridus 42010111002-0100100010·00003031112-111111203000·0203110000001···03002514111110100101????20

35. GON_Acanthopachylus_aculeatus 4101011000010100100010·000030311102111111203000·0200110000001···04002514111100112101100220

36. GON_Pachyloides_hades 4101011000010100100010·0000303111221[01]1111203000·0203110000001···04002514111100110101110021

37. GON_Eusarcus_hastatus 4000001300022121100011·000000311122111111203000·0203110000001···04002514111100110111100220

38. GON_Eubalta_meridionalis 4101011000012100100000·010030311102111111203000·0203110000001···04002514101100110111100220

39. GON_Acrographinotus_sp. 4101012010000100100000·010030311112111111203000·0203110000001···03002514111022110111110212

40. GON_Acutisoma_longipes 42010131002-0100100013·010000111120101111203000·0203110000001···03002514101100100101100211

41. TRIC_Tricommatus_brasiliensis 2001011000000000100000·000000?10002--0101203100·0200011100001···040025????110010-110110032

42. TRIC_Tricommatus_giuponii 2100011000000100100000·000000311121110101203100·0200011100001···0300251411112010-1?0210032

43. TRIC_Caramaschia_singularis 21?1111000000100?00302·0000003-0001--1101203100·020011110000?···040025--??11001012?0210032

44. Otilioleptes_marcelae 2101012000000000100010·000020000000001101203000·0000110000000···07002514111022004010200211

45. Osornogyndes_tumifrons 6101000000020021200300·010030000002001111203000·000311000000?···04202514111100112110110101
