# Supplementary material for: A relictual troglomorphic harvestman discovered in a volcanic cave of western Argentina: Otilioleptes marcelae, new genus, new species, and Otilioleptidae, new family (Arachnida, Opiliones, Gonyleptoidea)
Source: PLoS One. 2019 Oct 23;14(10):e0223828. doi: 10.1371/journal.pone.0223828 (PMC6808334; doi:10.1371/journal.pone.0223828)
Supplement: S1 Text — (PDF) [file pone.0223828.s002.pdf]

**S1 Text.**

**Characters, character states and coding, as applied in the matrix of S2 Table.**

Definitions, numbering and states follow Kury & Villarreal (2015), except for a few modifications (indicated as **), consisting in the addition of states (#8, 66, 67), or redefinition of characters (#52, 53) or states (#40). Characters #23, 48, 62, 63, 64 were disregarded, but their original numeration was kept (blank entries in the matrix). Characters #76 to #90 are new.

**• 1. DS (dorsal scutum), main outline in dorsal view**

0: eta (abdominal part size comparable to carapace, steadily growing wider posteriorly, with broad, convex posterior margin)
1: epsilon (subrectangular with corners rounded, no constrictions and slightly arched laterals)
2: alpha
3: beta (constricted twice, with coda, widest part on the posterior third)
4: gamma
5: zeta [similar to alpha (widest part in the middle), but narrower, with constrictions less extreme]
6: theta (bell-shaped, without posterior constriction, narrowing gradually)
7: delta
8: iota (subrectangular)

**• 2. Posterior margin of DS, shape**

0: convex
1: straight
2: concave

**• 3. Carapace outline, sexual dimorphism**

0: the same size in both sexes
1: much enlarged in males, groove projected backwards, squeezing scutal area I

**• 4. Ocularium, structure**

0: high coarsely granular, slanted forward
1: low, may have even median depression

**• 5. Placement of the eyes on two separate eyeballs (absence of a common eye mound), presence**

0: eyes placed on a common ocularium

1: two separate eyeballs

• **6. Frontal hump of carapace, presence**

0: absent

1: present

• **7. Scutal area I, presence of a median longitudinal groove**

0: absent, area I entire

1: present, area I divided into left and right halves

2: secondary effacement

3: *Goniosoma*-like (area II invading area I)

• **8. Scutal area III, paired armature** **[state 3 added to K&V 2015]

0: unarmed

1: with paramedian pair of tubercles or spines

2: high spines, partially fused

3: one median apophysis

• **9. Median spiniform apophysis of free tergite III, shape**

0: absent

1: stout

2: small

• **10. Median spiniform apophysis of free tergite II, presence**

0: absent

1: present

• **11. Furrow between scutal areas III and IV, degree of effacement**

0: furrow complete

1: furrow effaced in the middle

2: furrow entirely effaced

• **12. Furrow between areas III and IV, shape**

0: straight

1: clearly recurved (pointing forwards)

2: gently procurved (pointing backwards)

3: barely distinct, sinuous-formed by two joined Cs-clearly marked, sinuous

4: entirely effaced

• **13. Lateral margins of dorsal scutum, armature**

0: finely granular or without special granulation arranged in a row

1: with longitudinal row of light colored large tubercles, coalescent and flattened

2: with a marginal row of light colored tubercles well spaced between them

- **14. Stigmatic area, posterior border**
  - 0: straight or only very slightly concave
  - 1: deeply concave
- **15. Basichelicerite, structure**
  - 0: short, unarmed
  - 1: pre-bulla very long, powerfully armed
  - 2: bulla long, robust, with marginal armature
  - 3: bulla of male attenuate
  - 4: long and very thick
- **16. Posterior margin of cheliceral bulla, ornamentation**
  - 0: smooth
  - 1: with several teeth
- **17. Cheliceral hand of male, sexual dimorphism**
  - 0: present, cheliceral hand balloon-like swollen
  - 1: absent, cheliceral hand similar in both sexes
  - 2: intermediate, chelicera of male a little larger
- **18. Cheliceral fingers, elaborate dentition, presence**
  - 0: absent, teeth reduced and subequal
  - 1: present, teeth conform to varied categories, fingers often twisted and bent
- **19. Pedipalpal trochanter, modification for locking**
  - 0: without modifications
  - 1: with ventro-basal apophysis interlocking against cavity in coxa I
- **20. Pedipalpal femur, shape**
  - 0: cylindrical
  - 1: compressed, slightly concave mesally, with ventral row of numerous small spines coalesced into a flap
  - 2: compressed, with ventral row of independent small spines
  - 3: slightly compressed, accommodated wrapping chelicerae
- **21. Pedipalpal femur, disto-mesal setiferous tubercle. presence**
  - 0: absent
  - 1: present

- **22. Pedipalpal femur, ventral row of spines**
  - 0: absent
  - 1: numerous short teeth
  - 2: few stout spines
  - 3: one basal larger and a few smaller in the middle
- 23. (disregarded)
- **24. Pedipalpal tibia, shape**
  - 0: semi-cylindrical, without flap
  - 1: strongly depressed and concave, all setae fused forming a marginal flap
- **25. Pedipalpal tibia, ventro-ectal setiferous tubercles, size relationship**
  - 0: subequal
  - 1: penultimate or last much larger
- **26. Pedipalpal tarsus, shape**
  - 0: large, subrectangular, with normal claw
  - 1: small, subtriangular with claw much reduced
- **27. Patella and tibia of pedipalp, granulation of dorsal surface**
  - 0: finely granular
  - 1: with coarse tuberculation
- **28. Pedipalp, general size compared to the body**
  - 0: as long as scutum
  - 1: extremely elongate and thin
  - 2: robust, clearly larger than scutum
  - 3: stunted, shorter than scutum and thin
  - 4: not comparable, cosmetiform
- **29. Tarsus I, basal articles fused forming a spindle**
  - 0: absent
  - 1: present
- **30. Relative thickness of legs I–IV**
  - 0: all subequal
  - 1: III–IV clearly stronger than I–II
  - 2: legs getting steadily stronger from I to IV
  - 3: only leg IV much stouter than the others in the males

- **31. Coxa IV, dorso-apical apophysis only in male**
  - 0: absent
  - 1: present
- **32. Coxa IV of male, armature**
  - 0: weakly developed, armed with small spine or unarmed
  - 1: very robust horn-like apophysis
- **33. Coxa IV of male, position relative to dorsal scutum in dorsal view**
  - 0: concealed under DS
  - 1: clearly surpassing DS
- **34. Trochanter IV of male, retro-distal region, armature**
  - 0: unarmed
  - 1: stout recurved hook
  - 2: short spiniform apophysis
  - 3: stout procurved hook
- **35. Femur IV of male, structure**
  - 0: elongate, thin, cylindrical, sub-straight
  - 1: short, slightly sinuous, thickened
  - 2: moderately long, only slightly incrassate sinuous
- **36. Femur IV, sexual dimorphism**
  - 0: absent
  - 1: marked
- **37. Tibia IV, sexual dimorphism**
  - 0: absent
  - 1: marked
- **38. Distitarsus of leg I, segmentation**
  - 0: with 2 articles
  - 1: with 3 articles
- **39. Tarsal claw, legs 3–4, structure**
  - 0: median prong, with lateral secondary prongs
  - 1: paired claws
- **40. Tarsal process (Roewerian 'pseudonychium')** [K&V 2015, state #1 redefined]
  - 0: absent

1: present, either vestigial, short or long

2: present, very thick

• **41. Truncus penis, presence of musculature**

0: present, one muscle along the shaft

1: absent, gland moved by hydraulic pressure

• **42. Pars distalis of truncus penis, structure**

0: Distal region of truncus bears MS, but it is not clear-cut defined, being continuous with pars basalis

1: Ventral plate (VP) present in the form of a lamina parva, separated from the base by a waist

2: completely defined as a flattened subrectangular VP, clearly separated from truncus

3: Truncus uniform, topped by a tongue-shaped setigerous plate, homology with macrosetae of other Laniatores is uncertain.

4: VP well-defined, immensely developed, complex-shaped, separated from truncus by a neck

• **43. Staff-like break on distal portion of truncus, presence**

0: absent

1: present

• **44. Division of distal setigerous region of truncus**

0: complex system of plates detached from truncus

1: gradual narrowing of apex without distinction of a podium or a VP

2: Lamina parva well-marked, but extending dorsal – podium absent

3: distal truncus well-cut as a podium and VP well-marked

• **45. Ventral plate (VP), position in relation to truncus**

0: in the same plan

1: bent upon VP

• **46. VP, length**

0: long, at least twice as long as truncus width

1: short, as long as truncus width

• **47. VP, insertion in truncus apex**

0: not sunken into truncus

1: sunken into truncus

• 48. (disregarded)

• **49. Metasarcine spiny sacs, presence**

0: absent

1: present as a pair of haematodochae latero-subdistally on truncus penis

• **50. VP, overlapping with distal truncus**

0: not overlapping

1: truncus reaching about half-length of VP

2: truncus overlapping very little, only at base

• **51. Ampycus-like VP, oval, with deep cleft on distal border, presence**

0: absent

1: present

• **52. Glans penis, dorsal border** **[redefined from K&V 2015]

0: gentle transition from glans to stylus (straight or slightly curved)

1: presence of dorsal process, thumb-like

2: laminar as a keel

3: glans dilated dorsad, stylus arising perpendicular

• **53. Glans penis ventral process, presence** **[redefined from K&V 2015: only presence/absence is coded here; VPS type is considered in #81]

0: absent

1: present

• **54. Glans, shape**

0: embodied in the capsula externa

1: as a haematodocha, relatively free in the apical part of truncus

2: mounted atop the follis

• **55. Glans complex, size and position in relation to VP**

0: stylus short, atop a short glans

1: stylus long, in situ surpassing VP because situated on a long podium + glans,

2: stylus very long, atop a short glans, in situ surpassing VP

3: stylus elongate, upon a long column, surpassing VP

4: stylus very short, atop a triangular glans

• **56. Sac glans, structure**

0: non-columnar

1: columnar

• **57. Basal glans sac, structure**

0: formed by amorphous folds

1: formed by superimposed rings

• **58. Stylus, distal portion, shape**

0: cylindrical, or otherwise non-compressed

1: somewhat compressed, with small serrate crest and ill-defined carena

2: clearly compressed, with well-developed ventral serrate crest and well-marked carena

• **59. Stylus, structure**

0: cylindrical, with terminal opening

1: strongly flattened and expanded as a serrate keel, with opening as a slit displaced to dorsal

2: Metalibitia-like – sui generis

• **60. Stylar cap, presence**

0: absent

1: present

• **61. Ventral plate or ventral surface of penis, microsetae cover**

0: glabrous, microsetae absent

1: covered by one or two fields or isolated clumps of microsetae of varied shapes

• 62. (disregarded)

• 63. (disregarded)

• 64. (disregarded)

• **65. Macroseta A, position**

0: inserted on lateral border of VP

1: encircling VP as a girdle

• **66. Macroseta A-B, size and arrangement ****[state 7 added to K&V 2015]

0: all fairly elongate arranged as a girdle, B more ventral

1: lacking entirely

2: A1–A2 forming slanted row, with B extremely reduced

3: all relatively sturdy, forming an arch, B a little weaker, always positioned latero-ventralmost in the arch

4: all a little reduced in size and number, B proximal to A, lateral or ventro-lateral-two A and 1 B forming a triangle, with A lateral in a longitudinal row and B slightly shifted to latero ventral

5: both A and B inserted far distally, all robust and long. B lateral to ventro-lateral.

6: Macroseta A taking part in a equal gap row with C on laterals of VP

7: Basal group of numerous, stout and transverse spines (*Otilioleptes*)

- **67. Macroseta A, position** **[state 2 added to K&V 2015]
  - 0: more proximal, with gap between A and C
  - 1: more distal, adjacent to C
  - 2: group A split (one basal, the rest distal, adjacent to C)
- **68. Macroseta B, presence**
  - 0: present
  - 1: absent
- **69. Macrosetae C, basic placement**
  - 0: composing with A an outer girdle to glans, outside D
  - 1: not as a girdle, neither aligned with A, but still dorso-lateral on VP
  - 2: as a longitudinal lateral row distal on VP
- **70. Macrosetae C, shape**
  - 0: short, sturdy, at least partly lanceolate
  - 1: robust, with point lanceolate
  - 2: extremely long and slender
  - 3: very long and flattened
  - 4: robust, buffalo-horn
  - 5: moderately long and twisted, slender, acuminate
- **71. Macrosetae D**
  - 0: 1 pair, inserted fully dorsal, at the midway sides of the glans
  - 1: 1 pair, inserted much more distally on dorsal surface of VP, shifted more to lateral
  - 2: 1 or 2 pairs, well developed, shifted to distal, but still clearly dorsal
  - 3: 2 pairs, very small, dorso-basal near the glans
  - 4: 2 pairs, forming a mid-dorsal comb, located basal to glans
- **72. Macrosetae D, insertion**
  - 0: 1 to 3 pairs fully dorsal, surrounding glans, 1 pair at least as base of glans
  - 1: only one pair, migrated to latero-dorsal among A-C

2: one pair dorso-lateral at base of glans
3: 1 or 2 pairs, fully dorsal, much distal
4: 1 pair lateral between A and C

• **73. Macrosetae E, position**

0: 2 pairs, entirely on the ventral surface of VP, forming a square
1: 2 to 4 pairs, strongly reduced, inserted on the latero-distal flange of VP

• **74. Macrosetae E, size**

0: large, size comparable to A, B or C
1: minute, size much smaller than A, B or C

• **75. Macrosetae, pattern**

0: pattern A–E not recognizable. Not organized in 5 groups and distributed around the VP surface. Homology obscure.
1: pattern A–E clearly recognizable within the A, B, C, D, E chaetotaxy.

<u>CHARACTERS ADDED</u>

• **76. Number of setae A/B**

0: multiple
1: basic A1A2+B, one may be added
2: all disappeared or just one left

• **77. Orientation of setae A/B**

0: diagonal, pointing proximad
1: procumbent, pointing proximad
2: transverse, pointing to the sides

• **78. Insertion of setae A/B**

0: all or most setae in the VP, at the truncus/VP boundary or more distally
1: in the swollen base of VP (malleus)
2: displaced to the truncus, i.e., clearly more basal than the truncus/VP boundary

• **79. Position of setae C**

0: displaced basad, up to second one third of VP
1: subterminal or terminal, not displaced
2: absent

• **80. Apical border of VP**

0: cleft

1: straight or slightly convex

2: gently concave

• **81. Stylus, form of VPS (complement of character #53)**

0: curved ventrad, either as flabellum or hook-like

1: filiform

2: straight - covered and bordered by apical spines

3: serrate funnel, arising straight from mid-stylus

4: straight - truncate

• **82. Size of frontal hump, relative to ocular mound**

0: equal sized as ocular mound

1: lower than ocular mound, or absent

2: taller than ocular mound

• **83. Coalescence of coxa IV and stigmatic area**

0: fully coalescing

1: the apical end of coxa IV is more or less independent

• **84. Border of stigmatic area, development**

0: wide (border exceeds coxa IV)

1: narrow

• **85. Pp coxa, development (ventral view)**

0: very short, hidden

1: subequal to Cx I

2: slightly elongated, surpasses Cx I

3: very elongated, pyramidal

• **86. Coxa II, shape and position (ventral view)**

In most species coxa II is markedly longer than coxa III, and its apical end is bent diagonally anteriad, be it forming an angle with the axis of coxa III (state 0) or smoothly curved (state 2); in a few cases, it is not bent and point almost laterally (state 2).

0: apical end diagonal

1: apical end curved

2: apical end not bent (coxa II sub-transverse)

• **87. Coxa I-II, relative length (ventral view)**

0: Cx I and II subequal (or CxII a little shorter)

1: Cx II longer

• **88. Coxa III, length relative to coxa II (ventral view)**

This feature is based on the identification of 3 landmarks: A (proapical angle of coxa I), B (proapical angle of coxa II) and C (retroapical angle of coxa II), all proyected perpendicularly towards the anterior border of coxa IV. Thereby the relative length of coxa III can be referred to its extent on areas A-B and B-C.

0: CxIII much shorter (much less than B)

1: CxIII moderately short (fills up B)

2: CxIII moderately long (surpasses B)

3: CxIII equals CxII (fills up C)

• **89. Male coxa IV, development (ventral view)**

0: short, brief, lateral

1: hyperthelic, diagonal

2: hyperthelic, globose

3: short, robust

• **90. Presence of ventro- retroapical apophysis on coxa IV**

0: absent

1: small

2: well developed
