# Supplementary material for: A relictual troglomorphic harvestman discovered in a volcanic cave of western Argentina: Otilioleptes marcelae, new genus, new species, and Otilioleptidae, new family (Arachnida, Opiliones, Gonyleptoidea)
Source: PLoS One. 2019 Oct 23;14(10):e0223828. doi: 10.1371/journal.pone.0223828 (PMC6808334; doi:10.1371/journal.pone.0223828)

S1 Figure. Summary of cladistic relationships of *Otilioleptes* gen. nov. and the main clades within Laminata (L), in the different analytical treatments performed in this paper. A: IW, k=1–5; B: IW, k=6–13; C: IW, k=14–15; D: symmetric resample (k=6); E: jackknifing (k=6); F: standard bootstrap (k=6); G: EW, strict consensus; H: EW: majority rule.

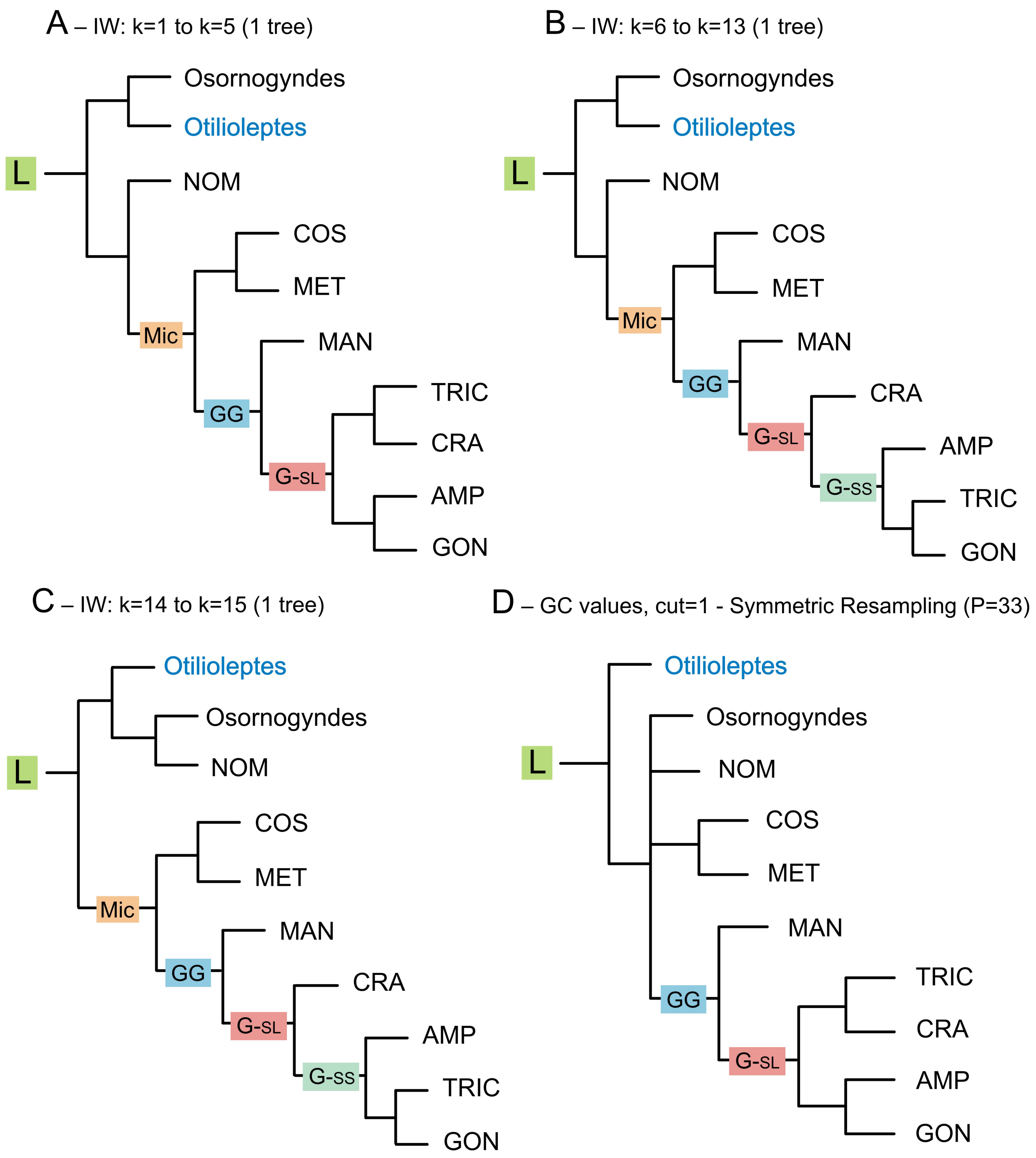

S1 Figure. Summary of cladistic relationships of *Otilioleptes* gen. nov. and the main clades within Laminata (L), in the different analytical treatments performed in this paper. A: IW, k=1–5; B: IW, k=6–13; C: IW, k=14–15; D: symmetric resample (k=6); E: jackknifing (k=6); F: standard bootstrap (k=6); G: EW, strict consensus; H: EW: majority rule.

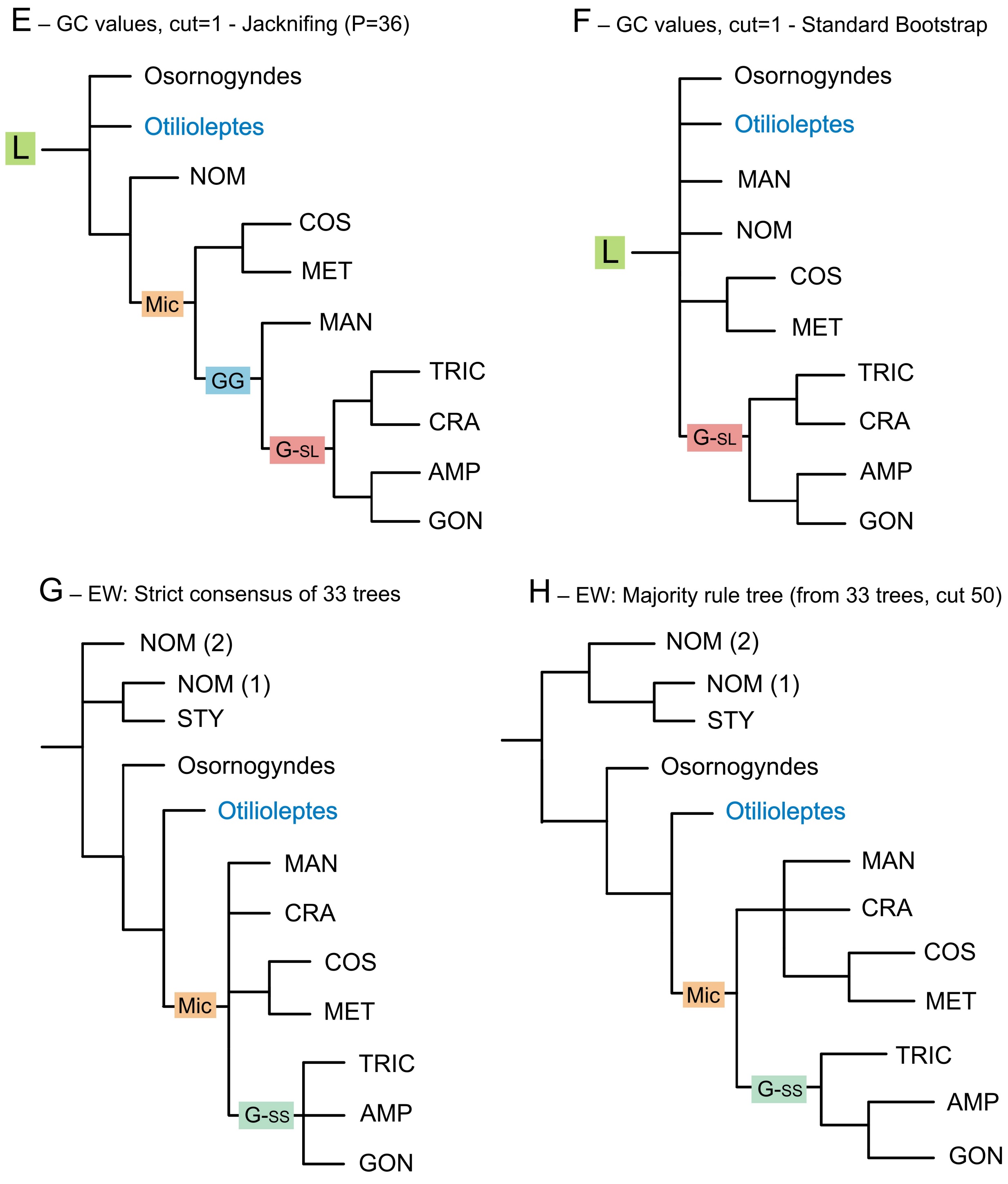

Supplement: S1 Fig — (PDF) [file pone.0223828.s003.pdf]
