# Supplementary material for: A relictual troglomorphic harvestman discovered in a volcanic cave of western Argentina: Otilioleptes marcelae, new genus, new species, and Otilioleptidae, new family (Arachnida, Opiliones, Gonyleptoidea)
Source: PLoS One. 2019 Oct 23;14(10):e0223828. doi: 10.1371/journal.pone.0223828 (PMC6808334; doi:10.1371/journal.pone.0223828)

S2 Figure. Cladistic relationships of Gonyleptoidea and *Otiloleptes marcelae* gen. nov., sp. nov.: Character optimization on the selected tree (IW, k=6). Solid circles indicate non-homoplasious states, open circles, homoplasious states; character numbers are above the circles, state numbers below.

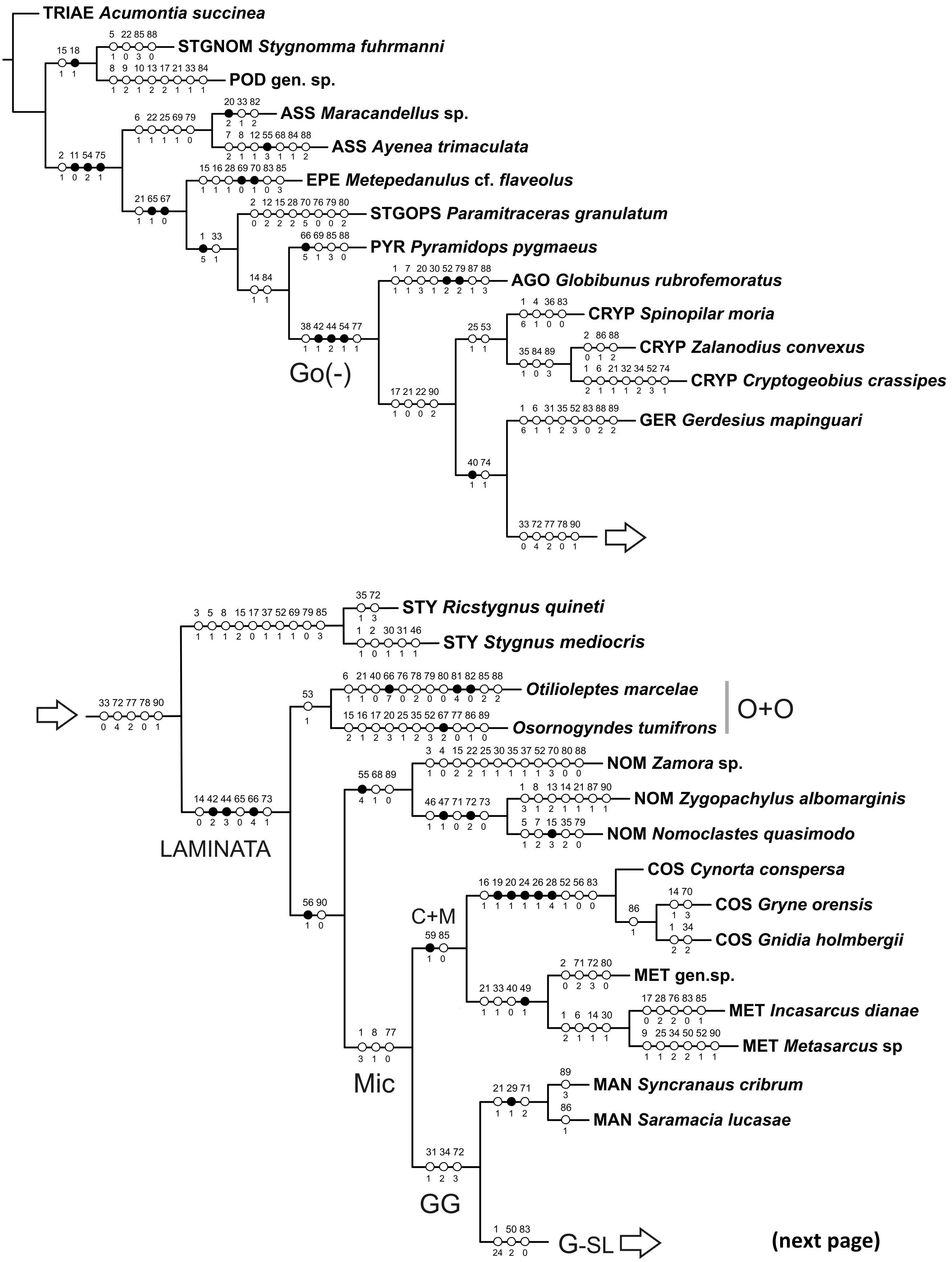

(next page)

S2 Figure. (continued)

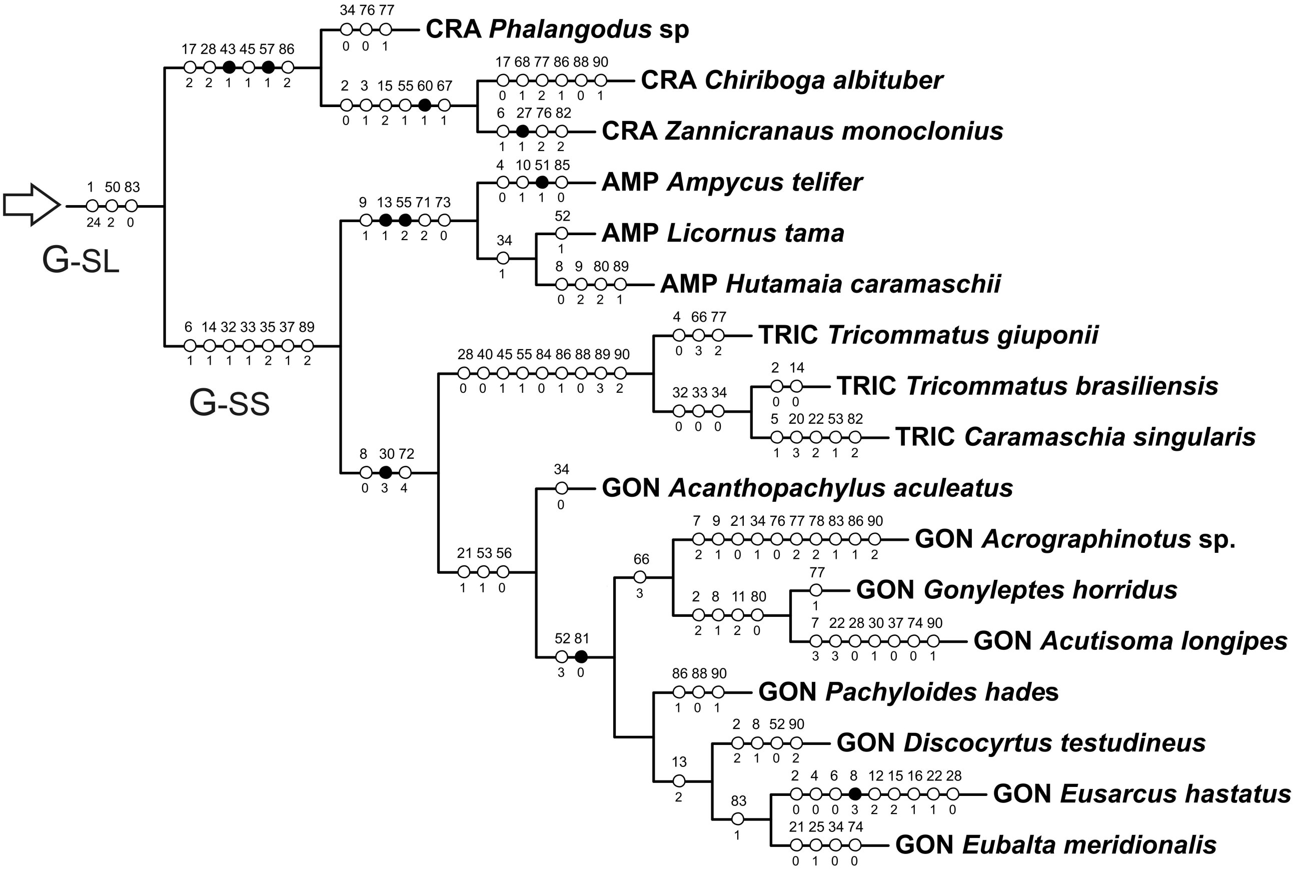

Supplement: S2 Fig — (PDF) [file pone.0223828.s004.pdf]
