# Supplementary material for: A relictual troglomorphic harvestman discovered in a volcanic cave of western Argentina: Otilioleptes marcelae, new genus, new species, and Otilioleptidae, new family (Arachnida, Opiliones, Gonyleptoidea)
Source: PLoS One. 2019 Oct 23;14(10):e0223828. doi: 10.1371/journal.pone.0223828 (PMC6808334; doi:10.1371/journal.pone.0223828)
Supplement: S3 Fig — (PDF) [file pone.0223828.s005.pdf]

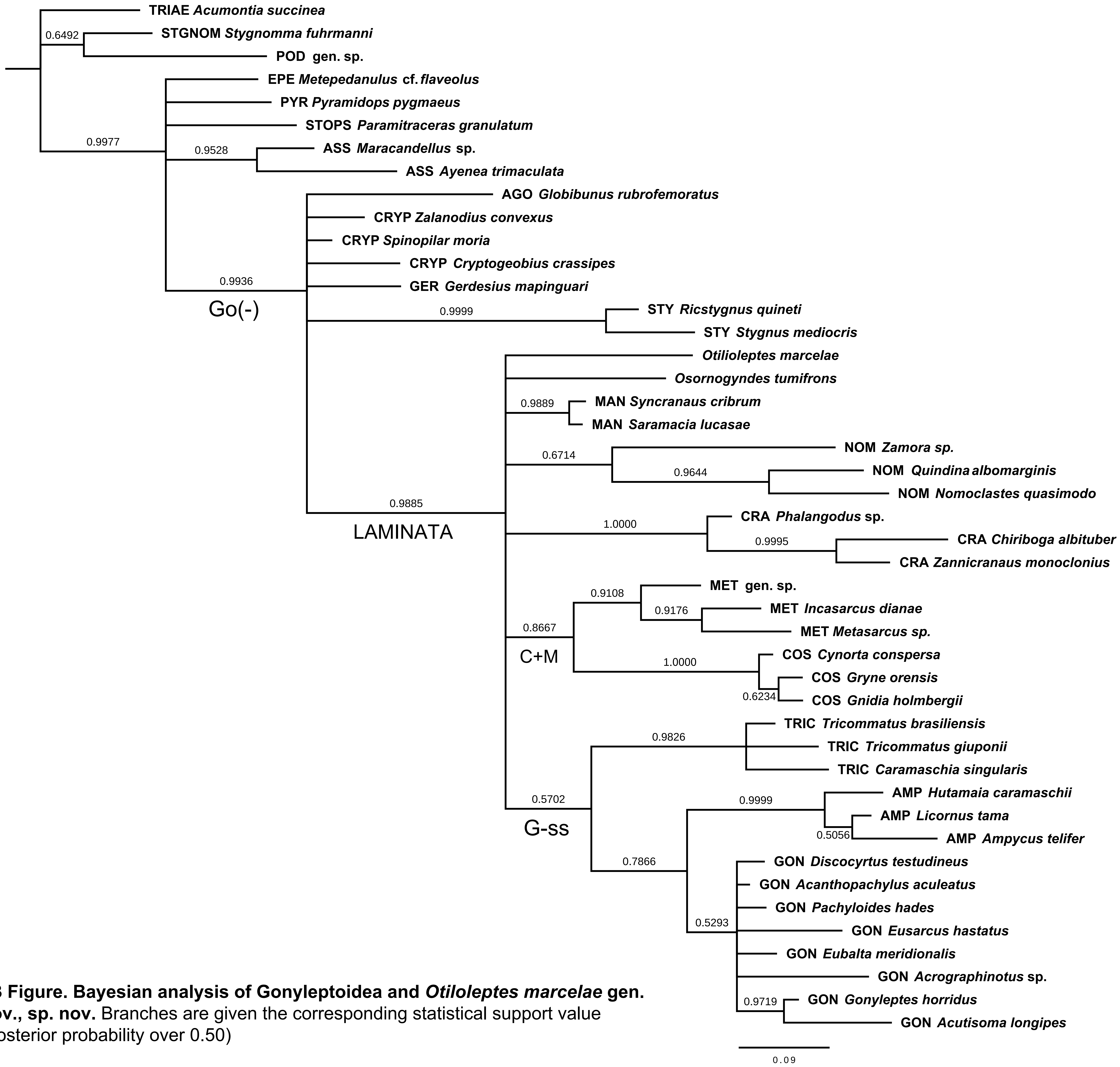

S3 Figure. Bayesian analysis of Gonyleptoidea and *Otiloleptes marcelae* gen. nov., sp. nov. Branches are given the corresponding statistical support value (posterior probability over 0.50)
